# A portable expression resource for engineering cross-species genetic circuits and pathways

**DOI:** 10.1038/ncomms8832

**Published:** 2015-07-17

**Authors:** Manish Kushwaha, Howard M. Salis

**Affiliations:** 1 Department of Biological Engineering, Pennsylvania State University, University Park, Pennsylvania 16802, USA; 2 Department of Chemical Engineering, Pennsylvania State University, University Park, Pennsylvania 16802, USA

## Abstract

Genetic circuits and metabolic pathways can be reengineered to allow organisms to process signals and manufacture useful chemicals. However, their functions currently rely on organism-specific regulatory parts, fragmenting synthetic biology and metabolic engineering into host-specific domains. To unify efforts, here we have engineered a cross-species expression resource that enables circuits and pathways to reuse the same genetic parts, while functioning similarly across diverse organisms. Our engineered system combines mixed feedback control loops and cross-species translation signals to autonomously self-regulate expression of an orthogonal polymerase without host-specific promoters, achieving nontoxic and tuneable gene expression in diverse Gram-positive and Gram-negative bacteria. Combining 50 characterized system variants with mechanistic modelling, we show how the cross-species expression resource's dynamics, capacity and toxicity are controlled by the control loops' architecture and feedback strengths. We also demonstrate one application of the resource by reusing the same genetic parts to express a biosynthesis pathway in both model and non-model hosts.

## Abstract (web summary)

Organism-specific genetic parts are often used to express circuits and pathways, limiting their portability. Here the authors engineer a cross-species expression resource, without using host-specific parts, to control protein and pathway expression in non-model bacteria.

## Graphical abstract

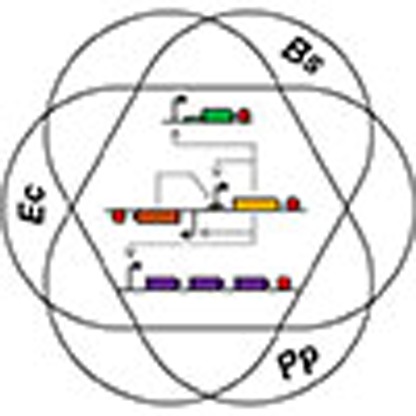

Engineered cell sensors, genetic circuits and pathways can reprogram cellular behaviour towards wide-ranging applications^1^,^2^,^3^, including the detection of cancerous cell states, digital and analogue computation, frequency multiplexing, memory storage and retrieval, the biosynthesis of valuable chemicals, and the remediation of toxic chemicals^4^,^5^,^6^,^7^,^8^,^9^,^10^,^11^,^12^. However, most advances in engineering genetic systems have so far been limited to a small number of model organisms, relying on host-specific regulatory genetic parts. Transferring a genetic system to a different organism alters the genetic parts' activities, often breaking the circuit or pathway behaviour^13^. A considerable amount of effort is then needed to rebuild the genetic system to adapt it to the new host's specifications^14^,^15^,^16^. Such incompatibilities are caused by the differences in gene expression machinery across species^17^,^18^,^19^,^20^, which have fragmented the fields of Synthetic Biology and Metabolic Engineering into host-specific domains where specialized genetic parts are tailor-made for each organism of interest^21^,^22^. To unify these efforts, a new paradigm is needed that enables one to use the same regulatory genetic parts in different hosts with similar functionality.

The portability of an engineered genetic system can be enhanced by minimizing its dependence on the native gene expression machinery and its cross-talk with competing cellular processes. Such insulation can be achieved by providing an alternative gene expression machinery that drives the engineered genetic system in a host-independent manner. Previous efforts to develop heterologous expression systems have utilized orthogonal viral polymerases together with their cognate promoter sequences for transcriptional insulation^23^,^24^,^25^,^26^. Alternatively, signal sequences have been identified that follow cross-species ‘consensus' rules that enable expression in a broad host range^27^,^28^,^29^. In both cases, previously developed heterologous expression systems have relied on the use of host-specific factors, for example, using an endogenous promoter to express T7 RNA polymerase (RNAP).

Instead, a portable genetic system cannot rely on host-specific promoters and the supply of its orthogonal gene expression machinery must be dynamically regulated to meet the demands of the engineered genetic system, while minimizing undesirable effects on the host. In particular, when overexpressing viral polymerases, the effect of cytotoxicity and unintended emergent phenotypes must be considered^25^,^30^,^31^. Cross-species portability can be achieved by building genetic circuitry that autonomously regulates the production of an orthogonal polymerase, creating a portable power supply that enables the same genetic system to be similarly expressed in different hosts, while using the same set of host-independent regulatory genetic parts^32^,^33^. The concept of a portable power supply for genetic systems is analogous to how electronic power supplies have been engineered to provide silicon circuits and computers with a near-constant voltage and amplitude of direct current even though their source of alternating current may be variable.

Here we describe the implementation of a type of ‘portable power supply' that achieves autonomously controlled levels of a viral RNAP across Gram-positive and Gram-negative bacteria. Specifically, the Universal Bacterial Expression Resource (UBER) combines host-independent transcription of an orthogonal T7 RNA polymerase, cross-species translation of messenger RNA (mRNA) and coupled positive and negative feedback loops (NFLs) to ensure auto-activation and self-limitation of the polymerase at nontoxic levels, and therefore similar expression outputs in diverse host contexts. We use computational biophysics-based design to rationally control the strengths of the feedback loops. By developing a system-wide mechanistic model alongside our experiments, we dissect the role of auto-activation, expression level dosage, competitive T7 RNA polymerase binding and feedback loop strengths on controlling the expression resource's dynamics, capacity and host toxicity. During this process, new design rules are formulated specifically for genetic systems that use orthogonal polymerases. Finally, we demonstrate how our expression resource enables cross-species metabolic pathway engineering.

## Results

### A cross-species gene expression system

**Figure 1:**
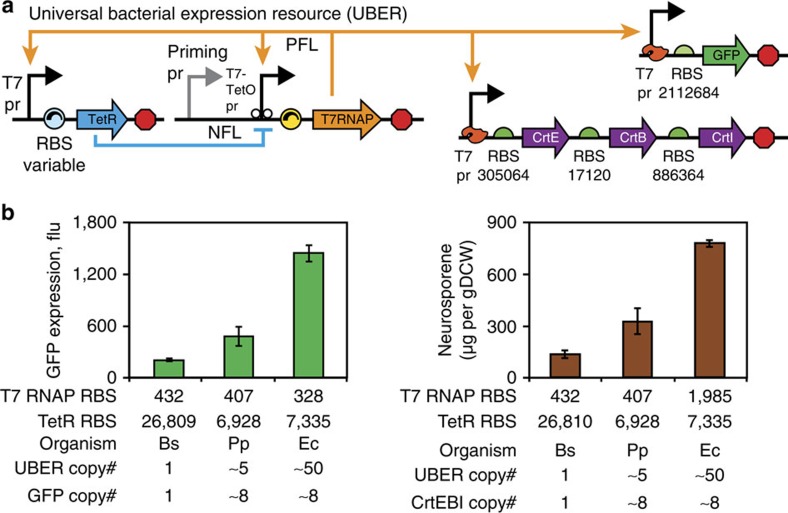
Genetic implementation of the Universal Bacterial Expression Resource for cross-species engineering. (**a**) The Universal Bacterial Expression Resource (UBER) consists of a mixed feedback loop (MFL) composed of two interlinked transcriptional feedback loops. The T7 RNA polymerase (T7 RNAP) drives an auto-activating positive feedback loop (PFL), while the TetR repressor controls the negative feedback loop (NFL). A core component of UBER is the T7 RNAP cassette that consists of a priming promoter (pr) for basal leaky transcription, a T7-TetO pr (T7 pr with flanking TetO operator sites) for controlling the PFL, a tuneable cross-species RBS, the T7 RNAP-coding sequence, and a set of intrinsic and T7 terminator sequences. The other core component is the TetR cassette that consists of a T7 pr, a tuneable cross-species RBS, the TetR-coding sequence and a set of intrinsic and T7 terminator sequences. The cross-species RBS is designed computationally to have similar translation rates in both Gram-negative and Gram-positive bacteria. The T7 RNAP produced in the system is used to drive the transcription of an output gene or operon of interest. RBS translation initiation rates were calculated using the RBS Calculator v2.0. (**b**) UBER achieves cross-species expression of a GFP reporter protein or a multi-enzyme pathway in (Bs) *Bacillus subtilis*, (Pp) *Pseudomonas putida* and (Ec) *Escherichia coli*. Here the enzymes CrtEBI synthesize the terpenoid neurosporene. The translation rates of the T7 RNAP and TetR ribosome-binding sites are shown alongside the copy numbers of the UBER and output modules. GFP fluorescence points and bars are the mean and s.d. of three measurements. Neurosporene content points and bars are the mean and s.d. of two measurements.

**Figure 2:**
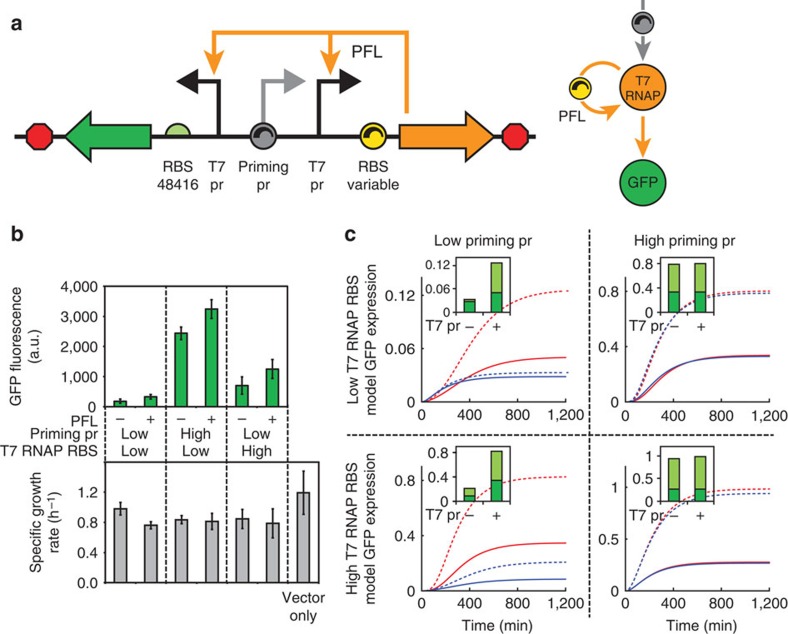
A positive feedback loop decouples expression capacity from host-specific transcription. (**a**) The T7 RNAP and the GFP cassettes of UBER are organized in divergent orientation on opposite DNA strands. The priming pr is a 456 bp DNA sequence of eukaryotic origin with 40% GC content that has low basal promoter activity in *E. coli*. Its basal activity can be increased by inserting a host-specific J23100 promoter upstream of the T7 pr. T7 RNAP PFL strength can be tuned by tuning the translation rate of the T7 RNAP RBS. (**b**) The top graph shows the average steady-state GFP fluorescences for UBER variants with and without the positive feedback loop, using either the (high) J23100 priming promoter or the (low) 456 nucleotides nonspecific promoter and either a high or low T7 RNAP translation rate. The bottom graph shows the corresponding specific growth rates of these UBER variants. Variants that lack (−) the positive feedback loop do not have a T7 pr driving T7 RNAP expression, causing T7 RNAP to be solely expressed by basal transcription from the priming pr. Variants with (+) the positive feedback loop use a T7 pr to self-amplify T7 RNAP expression. The predicted T7 RNAP translation rates are either low (97, 328, 97 and 91 a.u.) or high (7,656 and 8,378 a.u.) in left to right order. Data points and bars are the means and s.d. of three measurements. (**c**) Solid lines: time-course model solutions showing GFP levels corresponding to the characterized UBER variants both (red) with and (blue) without the positive feedback loop. Dotted lines: corresponding time-course model solutions if T7RNAP toxicity is not considered in the model. The model solution for the high/high UBER variant shows very high T7 RNAP expression levels; this UBER variant could not be successfully constructed. Inset: dark green, the steady-state GFP levels with and without the positive feedback loop are shown. Inset: light green, corresponding solutions if T7 RNAP toxicity is not considered in the model.

The genetic implementation of the UBER system combines expression of T7 RNA polymerase (T7 RNAP) and tetracycline repressor (TetR) within a self-regulating genetic circuit that uses a cross-species priming promoter, wild-type T7 promoters, an engineered T7 promoter flanked by TetO operator sites (T7-TetO promoter), cross-species translation signals and efficient transcriptional terminators (Fig. 1a). The T7-TetO promoter expresses T7 RNAP, creating a positive feedback loop (PFL) that becomes activated when a small amount of T7 RNAP is initially expressed. To provide this initial amount of T7 RNAP expression, we use the priming promoter, which is a 456-bp sequence of eukaryotic origin that contains many bacterial promoter-like elements (Supplementary Fig. 1). On the basis of our experimental dissection of the system's function, the final amount of T7 RNAP expression is largely insensitive to the transcription rate of this priming promoter, decoupling the binding specificity of the host's RNA polymerase from UBER's expression capacity (Fig. 2). Excess T7 RNAP expression can also lead to host toxicity that affects growth rates, and consequently other molecular behaviour^30^,^31^. Therefore, the expression of T7 RNAP is additionally regulated by a NFL, whereby a T7 promoter controls expression of TetR, which in turn binds to the T7-TetO promoter and represses further expression of T7 RNAP. The final amount of T7 RNAP-based expression is determined according to the strengths of these PFL and NFL.

Cross-species expression of the T7 RNAP and TetR also required rational design of their ribosome-binding sites and protein coding sequences. We employed multi-objective codon optimization to identify synonymous codons that would satisfy three criteria: maximizing translation elongation rates across both Gram-negative and Gram-positive bacteria, using *Escherichia coli* DH10B, *Pseudomonas putida* KT2440 *and Bacillus subtilis* 168 as examples; eliminating direct or inverted repeat sequences that promote genetic instability; and removing putative RNAse E- and RNAse III-binding sites to improve mRNA stability^34^,^35^,^36^,^37^,^38^. To control their expression levels in different bacterial hosts, we then employed the RBS Calculator v2.0's biophysical model of translation initiation to rationally design synthetic ribosome-binding site sequences^39^. The model's ability to perform cross-species translation rate predictions has been previously validated^5^. By combining the model's predictions with multi-objective codon optimization, we designed cross-species translation signals that provide similar targeted rates of translation across these diverse bacteria (Fig. 1a).

UBER acts as a portable power supply for cross-species engineering by supplying autonomously regulated amounts of T7 RNAP for transcription of genes controlled by T7 promoters. We measured its capacity using a T7 promoter to express either a green fluorescent protein (GFP) reporter or a three-enzyme terpenoid biosynthesis pathway in *E*. coli DH10B, *P. putida* KT2440 *and B. subtilis* 168; these strains feature substantially different plasmid compatibilities, metabolisms and growth conditions (Methods). While the copy number of the UBER system and the T7 promoter-driven output modules varied across hosts, we measured comparable levels of expression and metabolic production titers (Fig. 1b). In particular, UBER-driven expression of the pathway in *E. coli* DH10B resulted in a threefold higher product titer, compared with expression of the same pathway by either the P_lacO1_- or P_BAD_-inducible promoters in *E. coli* MG1655 (ref. 40). After transforming eight UBER variants into both *E. coli* and *P. putida*, the rank order of their GFP expression levels was also found to be preserved (Supplementary Fig. 2), indicating that the expression capacity of UBER is preserved among species with similar growth rates. The above data provide evidence that the host-independent UBER can be ported between unrelated species without loss of function.

In the following sections, we systematically dissect the contribution of each feedback loop, and vary its strength, to develop a system-wide, sequence-dependent dynamical model that explains how changes in UBER's genetic parts control its expression capacity. By this combined experimental and computational analysis, we provide a compelling example of how competitive binding, transcription factor titration, gene level dosage and growth rate inhibition work together to control genetic system function. Our analysis shows that simple mathematical models do not explain PFL auto-activation and NFL self-repression; however, a mechanistic model that accounts for these rarely considered interactions can explain how these feedback loops work together to control expression capacity.

### Expression auto-activation using feedback control

We first investigated how the PFL worked together with the priming promoter to control its auto-activation. Without a PFL, we expected that a priming promoter with a low transcription rate would not be sufficient to drive output module expression. Once the PFL is added, we expected that its gain or strength, as determined by the T7 RNAP's translation rate, would determine the minimum transcriptional threshold for auto-activation. If the PFL strength was low, the priming promoter must have a high transcription rate to ensure auto-activation. However, at a sufficiently high T7 RNAP translation rate, the output module's expression level would be high regardless of the priming promoter's transcription rate. To test our understanding of the system's response, we constructed versions of the UBER system that expressed the T7 RNAP and the GFP reporter in configurations with different priming promoters and used either an open-loop (PFL^−^) or closed-loop (PFL^+^) architecture; PFL^−^ system lacks the T7 promoter that runs the T7 RNAP PFL in the PFL^+^ system (Fig. 2a). We characterized these UBER variants in *E. coli* DH10B in extended steady-state cultures and measured both GFP reporter levels and host growth rates (Methods) (Fig. 2b).

Coincident with our hypotheses, we developed a quantitative model where we could vary the transcription rate of the priming promoter, the T7 RNAP's translation rate, and numerically simulate the dynamics of the PFL and the output module's expression levels (Supplementary Notes 1 and 2; Fig. 2c). The model has three sets of parameters: sequence dependent, measurement dependent and globally fit. In the first set, all of the model's translation rate parameters explicitly depend on the genetic system's ribosome-binding site sequences according to our RBS Calculator v2.0 model calculations, multiplied by a gene-specific proportionality constant that remains the same throughout this study. In the second set, the host's growth rates were directly measured and used in model calculations. Finally, the remaining model parameters (for example, promoter transcription rates, mRNA degradation rates and polymerase/repressor-binding affinities) were parameterized using all available data, but remained consistent throughout this study. For steady-state simulations, toxic stress from prolonged T7 RNAP expression was accounted for by reducing all translation rates in the model as the T7 RNAP's expression level was increased, according to a sigmoidal relationship (Supplementary Note 5).

According to the model's calculations, in the open-loop case (PFL^−^), the output module's expression levels will increase proportionally with increases in either the priming promoter's transcription rate or the T7 RNAP's translation rate. When both are increased, the output module's expression level will increase in a multiplicative fashion. Our experimental characterization supports the model's calculations, as augmenting the priming promoter with a stronger J23100 promoter or increasing the predicted T7 RNAP translation rate from 97 to 7,656 (RBS Calculator v2.0 units) resulted in increased GFP expression levels. However, combining these increased transcription and translation rates together resulted in a non-viable host, likely due to toxic levels of T7 RNAP.

To compare, when we add the PFL loop to the model, the GFP reporter expression levels will always be higher than in the open-loop case (PFL^−^). Lowering the priming promoter's transcription rate can still provide high GFP reporter expression levels so long as the PFL strength is sufficiently high (Supplementary Fig. 3). Our experimental results agree with these model calculations, showing that the presence of the PFL does indeed increase GFP reporter expression levels and that the PFL's effect was strongest when the priming promoter had a low transcription rate. These results show that a transcriptional PFL with a sufficiently high strength can effectively decouple a weak priming promoter's low transcription rate from the output module's expression level (Supplementary Fig. 3), thereby insulating UBER's expression capacity from changes in the host's endogenous transcriptional machinery.

Importantly, the host's growth rate and T7 RNAP toxicity were crucial to the model's solution in both PFL^−^ and PFL^+^ systems. The growth rate introduces first-order dilution terms that partly counteract the non-linear auto-activation controlling T7 RNAP expression. The toxicity function in the model reduces translation rates of all proteins in response to T7 RNAP accumulation. This allows the model to better estimate steady-state solutions that are substantially lower than those expected after a time-course simulation in the absence of toxicity (Fig. 2c). The difference between the two reflects the T7 RNAP toxicity in the system, as seen most markedly in the model solutions for the non-viable high priming/high T7 RNAP variants.

### Expression capacity is controlled by the feedback loop gain

**Figure 3:**
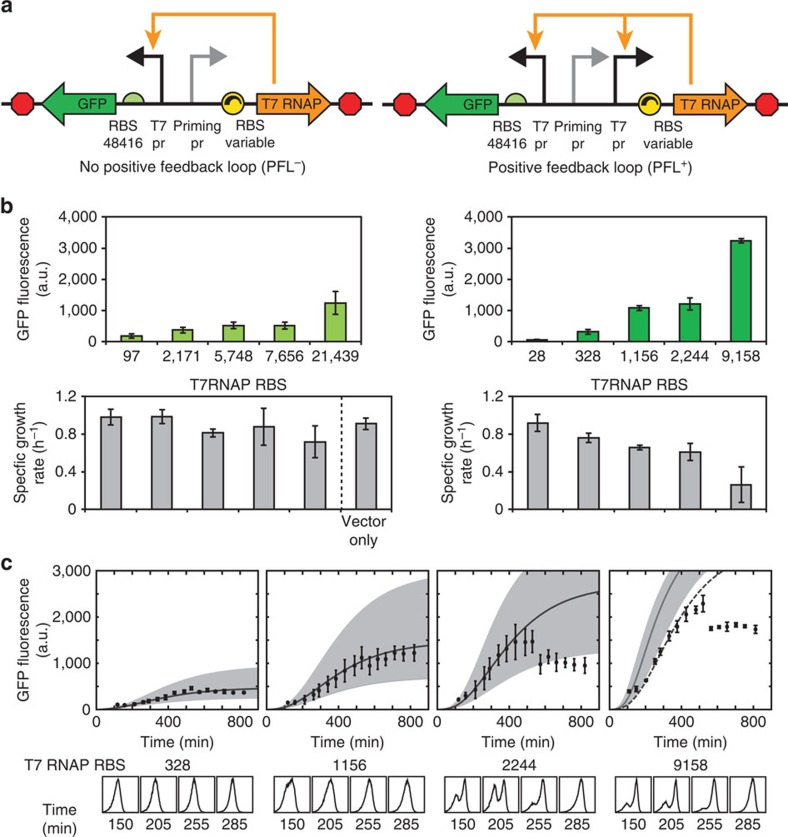
Auto-activation dynamics are controlled by the strength of the positive feedback loop. (**a**) Schematics of UBER variants with and without a positive feedback loop are shown. (**b**) Top: the steady-state GFP fluorescences and (bottom) host growth rates of UBER variants either with or without the positive feedback loop, while introducing T7 RNAP RBS variants with increasing translation rates. Data points and bars are the mean and s.d. of three measurements. (**c**) Top: A comparison between measured GFP expression levels and model dynamic solutions for four PFL(+) UBER variants with increasing T7 RNAP translation rates. The shaded region shows the model dynamic solution after a 2.2-fold change in translation rate prediction. Dotted line; the model dynamic solution for a translation rate prediction of 3,430 a.u. Bottom: single-cell GFP fluorescence distributions of the PFL(+) UBER variants at specified time points.

Next, we investigated how the quantitative strength of the PFL controls UBER's expression capacity and the host's growth rate. To systematically increase the effect of the PFL, we rationally designed synthetic ribosome-binding sites that increased T7 RNAP's translation rate from 28 to 9,158 a.u. (RBS Calculator v2.0 units). By increasing T7 RNAP's translation rate both in the open-loop and the closed-loop configurations (Fig. 3a), we were able to carefully dissect the PFL's role in controlling GFP reporter expression levels. According to our model, increasing T7 RNAP's translation has a twofold effect in the closed-loop configuration; it increases the initial amount of T7 RNAP expressed by the priming promoter, and it amplifies the amount of expressed T7 RNAP by the PFL's T7 promoter. In contrast, in the open-loop configuration, increasing the T7 RNAP translation rate only sublinearly increases GFP expression. These modelling results were well supported by our characterization of the system's expression capacity. GFP reporter expression levels increased by only 10-fold when increasing T7 RNAP translation rates by 200-fold; a very high T7 RNAP translation was needed to drive moderate GFP reporter levels (Fig. 3b). By closing the loop, GFP reporter levels increased substantially, while requiring lower T7 RNAP translation rates to achieve high output module expression levels. Results similar to *E. coli* were also obtained in *P. putida* and *B. subtilis* when PFL+ variants with tuneable T7 RNAP RBS strengths were introduced (Supplementary Fig. 4). However, this auto-activation at even moderate T7 RNAP translation rates led to excess expression of T7 RNAP and host toxicity, as measured by a significant reduction in the host's growth rate.

The apparent trade-off between PFL strength, expression capacity and host growth rate led us to further examine the dynamics of auto-activation. For several PFL UBER variants with different T7 RNAP translation rates, we characterized the dynamics of auto-activation, measuring the distribution of single-cell GFP expression over a 13-h period (Fig. 3c). We also simulated the dynamics of auto-activation using our quantitative model, directly feeding in the RBS Calculator v2.0 predicted translation rates without including the effects of T7 RNAP toxicity. The observed dynamics of auto-activation were well modelled by the simulation results, showing that the characterized auto-activation dynamics were initially controlled by the T7 RNAP translation rate according to the RBS Calculator v2.0 model. An exception occurred at a very high T7 RNAP translation rate, where the apparent translation rate was 2.6-fold lower than predicted, potentially due to T7 RNAP toxicity. Excess amounts of T7 RNAP can lead to nonspecific transcription of the genome, as well as overabundance of T7 RNAP and GFP transcripts, that sequester ribosomes and prevent translation of important housekeeping genes^41^,^42^,^43^.

### Differential partitioning of T7 RNAP in genetic circuits

**Figure 4:**
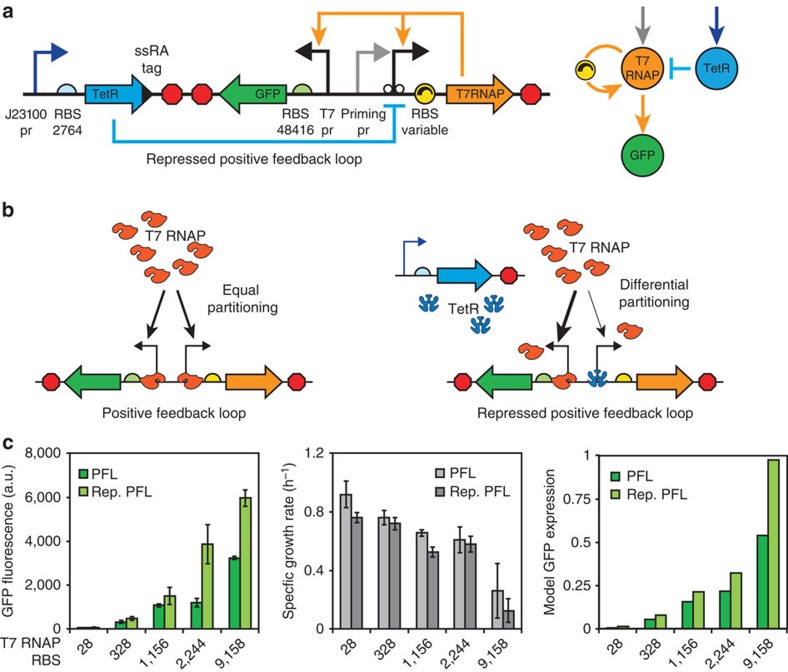
Competitive binding between TetR and T7 RNAP causes differential partitioning. (**a**) The repressed PFL version of UBER uses a constitutive J23102 promoter, a designed RBS, and an efficient transcriptional terminator to express TetR, which binds to the T7-tetO promoter and represses T7 RNAP expression. (**b**) Left: equal access to T7 promoters causes T7 RNAP to equally bind and partition between them. Right: when TetR is expressed, unequal access to T7 promoters causes T7 RNAP to differentially bind and partition among them. (**c**) Left: the steady-state GFP fluorescence for PFL(+) and repressed PFL UBER variants are shown with different T7 RNAP RBSs that have increasing translation rates. Middle: the measured growth rates for corresponding UBER variants are shown. Data points and bars are the mean and s.d. of three measurements. Right: a model including differential partitioning shows the simulated steady-state GFP expression levels for corresponding UBER variants.

Because uncontrolled auto-activation led to excess T7 RNAP levels and growth inhibition, we next investigated feedback mechanisms that would turn off auto-activation once a sufficient amount of T7 RNAP was expressed. We first used a J23102 constitutive promoter for expression of the TetR tagged with a C-terminal ssrA peptide to lower TetR's stability. In our original PFL system, expression of T7 RNAP is controlled by an engineered T7 promoter that contains two TetR-binding operator sites (T7-tetO). Therefore, when TetR is additionally expressed, it will bind to the T7-tetO promoter and reduce T7 RNAP expression, creating a repressed PFL genetic circuit (Fig. 4a).

According to the current design principles for engineering genetic circuits^1^,^3^,^44^,^45^, repressing T7 RNAP expression should also repress expression of the GFP output module. However, these rules were originally formulated for genetic circuits using native RNA polymerases and we expected key differences when using T7 RNAP. Specifically, when TetR is expressed and bound to the T7-tetO operator, there are fewer T7 promoters available to bind T7 RNAP, thereby increasing the binding occupancy of the remaining T7 promoters. In the repressed PFL system, the only remaining T7 promoter controls GFP expression, and therefore GFP's expression level could actually increase when TetR is expressed in contradiction to current design rules. Indeed, experimental characterization of the repressed PFL system showed that expressing TetR actually resulted in a significant increase in output module expression, as measured by GFP reporter levels (Fig. 4c).

The presence of TetR shifted the genetic circuit from equal partitioning of T7 RNAP across identical promoter sites to differential partitioning across different types of promoter sites (Fig. 4b). To quantify the differential partitioning of T7 RNAP, we expanded our model to account for the competitive binding of T7 RNAP and TetR to the T7-tetO promoter, the non-competitive binding of T7 RNAP to the T7 promoter controlling GFP expression, and mole balances constraining the amounts of T7 RNAP and TetR, both bound and unbound, within the system (Supplementary Note 3). According to the model, expressing TetR will increase the amount of unbound T7 RNAP, compared with the non-repressed PFL system, consequently increasing the GFP expression levels (Supplementary Fig. 5; Fig. 4C, right). The steady-state solution of the repressed PFL system with RBS 2244 shows that 60% of T7 RNAP copies are bound to a promoter; 99.4% of them are bound to the T7 promoter controlling GFP expression, while only 0.6% are bound to the T7-tetO promoter controlling T7 RNAP expression. The differential partitioning of T7 RNAP depends strongly on the T7 RNAP translation rate and the total amount of T7 RNAP expressed.

To test the model, we systematically increased the T7 RNAP translation rate from 28 to 9158 (RBS Calculator v2.0 units), characterized the repressed PFL circuit variants, and found that the model's differential partitioning mechanism is well supported by both growth rate and GFP expression measurements (Fig. 4c, left and middle). The host's growth rate was lower in the repressed PFL system, particularly at high T7 RNAP translation rates, as more T7 RNAP was unable to bind the T7-tetO promoter, and remained free to fortuitously bind elsewhere in the genome and contribute to host toxicity. At the same time, more T7 RNAP was available to bind the T7 promoter controlling output module expression, resulting in a significant increase in GFP reporter levels.

### Mixed feedback loops for subtoxic auto-activated expression

**Figure 5:**
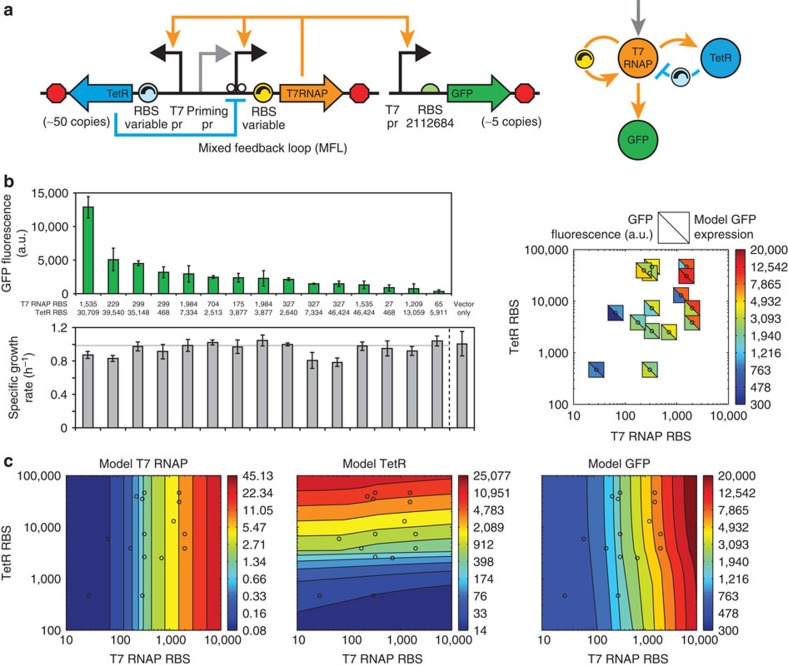
Systematic Variation of mixed feedback loop strengths eliminated T7 RNAP toxicity. (**a**) The mixed feedback loop (MFL) version of the UBER system expresses TetR cassette using a T7 promoter, a designed RBS, and an efficient T7 transcriptional terminator in a divergent orientation. The MFL strengths were varied by inserting optimized RBS libraries controlling the translation rates of the T7 RNAP and TetR. The MFL UBER system exists on a high copy (ColE1) origin. The output module expresses a GFP reporter on a lower copy plasmid (RK2 origin). (**b**) Left: the steady-state GFP fluorescences and measured host growth rates for the 15 characterized MFL variants are shown with different T7 RNAP and TetR RBS translation rates. Grey line: the mean growth rate is indicated. Right: squares represent comparisons between the measured GFP fluorescences and the model-calculated GFP concentrations for each MFL circuit variant across the range of T7RNAP and TetR RBS translation rates. Experimental points and bars are the mean and s.d. of three measurements. (**c**) Contour plots show the steady-state model solutions for the T7 RNAP, TetR and GFP concentrations across a range of T7RNAP and TetR RBS translation rates. Circles indicate the T7 RNAP and TetR RBS translation rates for the 15 MFL variants characterized.

In the repressed PFL system, TetR expression increased the output module's expression, but also decreased the host's growth rate, due to the accumulation of unbound T7 RNAP and its apparent toxicity (Fig. 4; Supplementary Fig. 5). We next investigated a mechanism to dynamically control TetR and T7 RNAP expression levels so that the amount of unbound T7 RNAP would remain low. To do this, we placed expression of the TetR repressor under control of a T7 promoter, using a TetR variant without an ssrA degradation tag. The resulting mixed feedback loop (MFL) genetic circuit combines interlocked NFL and PFL whose strengths were tuned by adjusting the translation rates of the TetR and T7 RNAP mRNAs (Fig. 5a). We also modified our quantitative model to account for these interactions and to calculate T7 RNAP, TetR and output module expression levels with different NFL and PFL strengths (Supplementary Note 4).

According to our mechanistic model, we expected to find that steady-state T7 RNAP and output module expression levels are largely controlled by the PFL's strength (Fig. 5c, left). The steady-state TetR expression levels are regulated primarily by the NFL's strength (Fig. 5c, middle). Consequently, the output module's expression level is controlled by the strengths of both feedback loops, highlighting the non-linear dynamics occurring within the mixed feedback system^46^ (Fig. 5c, right). The NFL begins to control the output module's expression only when T7 RNAP becomes highly expressed, triggering both expression of TetR and the differential partitioning of T7 RNAP towards expression of the output module. In particular, the NFL substantially reduces the amount of unbound T7 RNAP at all PFL strengths.

We constructed and characterized 15 variants of the MFL genetic circuit where we altered the strengths of the PFL and NFL by uniformly varying the translation rates of T7 RNAP from 27 to 1,984 a.u. and of TetR from 468 to 46,424 a.u. through the introduction of rationally designed RBS libraries, optimized by our RBS Library Calculator algorithm^5^. Using multi-fragment DNA assembly^47^, the two RBS libraries were combinatorially inserted into a plasmid-encoded MFL genetic circuit, followed by characterization of their GFP reporter levels and host growth rates. By incorporating a properly tuned NFL, we were able to significantly increase UBER's expression capacity by 10-fold and 4-fold, compared with the open-loop (PFL−) and closed-loop (PFL+) circuits, respectively, while eliminating T7 RNAP-specific inhibition of growth rate (Fig. 5b, left). Consistent with the model, the MFL variant with the highest T7 RNAP and TetR translation rates achieved the highest output module expression level. We also constructed two versions of the MFL combinatorial library using either TetR with or without an *ssrA* tag to lower its protein stability. We found that the MFL circuit using an untagged TetR variant, and therefore a higher accumulation of TetR, had overall higher output module expression levels (Supplementary Fig. 6), consistent with the model solution and in agreement with our previous results showing that higher steady-state expression of TetR results in increased GFP output, due to differential partitioning of T7 RNAP.

In further comparisons, we found that the MFL UBER circuits with different T7 RNAP and TetR translation rates had output module expression levels that were consistent with our model solutions for 11 of the 15 variants (Fig. 5b, right). Notably, the four MFL variants with larger divergences from the model solution are predicted to have high T7 RNAP levels, indicating higher toxicity effects (Fig. 5c). The mechanistic model enabled the accurate dissection of the MFL UBER system, capable of distinguishing between the effects of the two feedback loops, differential partitioning and T7 RNAP toxicity.

## Discussion

In the first decade of the Synthetic Biology field, genetic systems were primarily designed and constructed in model organisms, taking advantage of domesticated strains, vectors, markers, protocols and well-characterized genetic parts^1^,^2^. Replicating the development of such genetic tools and genetic parts in other novel organisms is time-intensive, costly and generally limited by the smaller community of researchers interested in using each organism, although there are exciting applications^8^. More recently, the development of CRISPR/Cas9-based genome editing has provided a versatile cross-species genome-editing technique^46^,^48^, enabling engineered genetic systems to be directly incorporated into genomes. Consequently, while we have the genetic tools to manipulate the genomes of many non-model organisms, we need new approaches to control cross-species gene expression levels using the same toolbox of well-characterized genetic parts.

In the past, cross-species control of gene expression depended on the evolutionary conservation of the host's machinery. It has been feasible to transfer ribosome-binding sites, transcriptional terminators, small RNAs, riboswitches and ribozymes^49^,^50^,^51^,^52^,^53^,^54^,^55^ from one bacterium to another because their functions largely depend on well-conserved RNA–RNA and RNA–ribosome interactions. In contrast, a promoter's transcription rate depends on protein–DNA interactions, and these interactions are not well-conserved across homologous sigma factors, RNA polymerases and transcription factors^17^,^56^. As a result, it is far more difficult to design promoters with both broad host specificities and high transcription rates^19^.

Here we have developed a solution to overcome the cross-species challenge by engineering a UBER that autonomously self-regulates the expression of an orthogonal T7 RNA polymerase, without using host-specific promoters, enabling genetic circuit and metabolic pathway engineering in diverse Gram-negative and Gram-positive bacteria (Fig. 1a). We demonstrate that identical multi-enzyme terpenoid biosynthesis pathways, expressed by UBER, function similarly across different organisms (*E. coli*, *B. subtilis* and *P. putida*) even though their native promoters are starkly different (Fig. 1b). UBER is the first example of a portable power supply for cross-species genetic system engineering.

We then constructed and characterized 50 variants of the UBER system to dissect its self-regulatory control properties, systematically perturbing its feedback loop strengths and combining experimental measurements with mechanistic modelling to understand its emergent behaviour. We show that the PFL is essential for achieving host promoter independence (Fig. 2) and that the feedback loop strength directly controls the output module's expression dynamics and capacity (Fig. 3). We present a new design principle that explains why, when using orthogonal polymerases, the transcriptional regulation of one promoter can affect the transcription rate of other promoters (Fig. 4). Finally, we show how the interplay between the PFL and NFL eliminates T7RNAP-dependent growth inhibition, while enabling one to tune the expression capacity of the UBER system (Fig. 5).

The cross-species capability of UBER may be readily combined with recent advances in engineering orthogonal polymerases and promoters. First, modifying the T7 RNAP promoter sequence provides fine control over its transcription rate^25^. Using this strategy, we additionally show that such promoters can be combined with UBER to rationally control the output module's transcription rate (Supplementary Fig. 7). Further, split versions of T7 RNA polymerase have been developed to enable digital logic and transcriptional resource allocation in *E. coli*^26^,^57^,^58^. Directed evolution has also been applied to T7 RNAP to generate variants with varying promoter-binding affinities and activities^59^. Such T7 RNAPs could be readily integrated into our MFL genetic circuit to autonomously self-regulate their expression and enable their use in both model and non-model hosts. Furthermore, UBER's portability can be expanded to bacteria with highly divergent anti-Shine–Dalgarno sequences by implementing a staggered RBS design strategy (Supplementary Fig. 8).

Overall, UBER accelerates the prototyping of genetic circuits and metabolic pathways in non-model hosts without requiring the characterization or reuse of native host promoters. Several industrially important bacterial strains, such as *Bacillus*, *Pseudomonas*, *Clostridium* and *Lactobacillus*, lack the well-characterized collection of promoters found in *E. coli*, although modifying their metabolism may be particularly advantageous to overproducing a desired chemical or natural product^8^,^60^,^61^,^62^,^63^,^64^,^65^. Decoupling the host's metabolism from its gene regulation is necessary when engineering metabolic pathways to overproduce a desired product, while still relying on the host's biosynthesis of essential cofactors and metabolites. Instead of limiting host selection, UBER enables metabolic pathways to be expressed and prototyped across such non-model, but tractable, organisms. Similarly, when elucidating the pathway for a natural product in a non-model host, UBER enables the expression of modified or refactored gene clusters without interference from native gene regulation^66^.

As a result, portable power supplies such as the UBER will markedly increase the modularity, reusability and higher-level abstraction of engineered genetic systems. We envision that by the end of the second decade of Synthetic Biology, such portable power supplies will increasingly blur the boundaries between model and non-model hosts, and eliminate the limitations of relying on host-specific genetic part toolboxes. Instead, the design of engineered genetic systems will be decoupled from the host's transcriptional machinery, enabling increased sharing of genetic modules between groups of researchers exploring a wider range of biotechnological applications.

## Methods

### Bacterial strains and culture conditions

*E. coli* DH10B, *P. putida* KT2440 (ATCC# 47054) and *B. subtilis* 168 (BGSCID# 1A1) used in this study were cultured in LB broth Miller (10 g l^−1^ tryptone, 5 g l^−1^ yeast extract and 10 g l^−1^ NaCl) obtained from BD (#244610). Solid media for plates was prepared in all cases by adding 1.5% w/v agar (BD# 214010). In addition, 1% w/v potato starch (Sigma-Aldrich# S5651) was added to the solid media for *B. subtilis* plates. *E. coli* cells were grown at 37 °C, while *P. putida* and *B. subtilis* were grown at 30 °C.

Antibiotic selection used for *E. coli* single-plasmid UBER system was 30 μg ml^−1^ ampicillin (Teknova# A9509), while that for the dual-plasmid system was 30 μg ml^−1^ ampicillin+30 μg ml^−1^ kanamycin (EMD Millipore# 420311). Antibiotic selection used for *P. putida* single-plasmid UBER system was 30 μg ml^−1^ kanamycin, while that for the dual-plasmid system was 30 μg ml^−1^ kanamycin+150 μg ml^−1^ chloramphenicol (Alfa Aesar# B20841). *B. subtilis* cells with single-genomic integration were selected at 5 μg ml^−1^ chloramphenicol (Alfa Aesar# B20841), while those with double-genomic integration were selected at 5 μg ml^−1^ chloramphenicol+3 μg ml^−1^ kanamycin.

To make them electrocompetent, diluted overnight cultures of *E. coli* and *P. putida* were grown in an incubator shaker (300 r.p.m.) at 30 °C to mid-exponential phase OD_600_ of 0.4–0.6, washed two times in ice-cold 10% v/v glycerol and finally resuspended in chilled 20% v/v glycerol. The cells were then transformed by electroporation using an Eppendorf Electroporator-2,510 at 1,800 V. *B. subtilis* cells were made competent by first growing a diluted overnight culture in MD medium (+20% casamino acids) in an incubator shaker (300 r.p.m.) at 37 °C to an OD_600_ of 1.0–1.5, and then diluting the culture by 2 × in fresh MD medium (no casamino acids) and growing for an additional 1 h. They were transformed by incubation with DNA for 20 min, followed by addition of 20% casamino acids and 1-h long recovery^67^. Following selection, *B. subtilis* colonies were screened for correct genomic integration into the *amyE* locus by the iodide starch plate assay, and into the *lacA* locus by colony PCR.

### Plasmid construction

The optimized coding sequences (Supplementary Table 1) were synthesized by GeneArt, and the various plasmids were constructed using standard cloning techniques involving PCR, primer annealing, restriction digestion, gel extraction, ligation, as well as chew-back anneal-repair^47^. All cloning was performed in *E. coli* DH10B, and constructs were confirmed by sequencing before retransformation into the relevant species.

Single-plasmid UBER variants of *E. coli* were constructed on the pPM47 plasmid backbone^68^ (Addgene plasmid 20132) after removing the mCherry insert and the T7 promoter outside the multiple cloning site (*ampR*, ColE1 replication origin). For the *E. coli* dual-plasmid variants, the *T7RNAP* and *tetR* cassettes of the MFL system were similarly cloned on the pPM47 plasmid backbone, and the *gfp* or the *crtEBI* cassette was cloned on a modified pAKgfp1 plasmid backbone with RK2 replication origin^69^ (Addgene plasmid 14076) after removing the *gfp* insert and replacing the *ampR* gene with the *kanR* gene. Single-plasmid UBER variants of *P. putida* were also cloned on the modified pAKgfp1 plasmid backbone (*kanR*, RK2 replication origin). For the *P. putida* dual-plasmid variants, the *T7RNAP* and *tetR* cassettes were cloned on the modified pAKgfp1 plasmid backbone (*kanR*, RK2 replication origin), and the *gfp* or the *crtEBI* cassette was cloned on pSEVA351 vector with oriT replication origin (GenBank Accession JX560335, *cmR*). These dual-plasmid *P. putida gfp* variants (RK2, oriT) were also tested in *E. coli* to compare cross-species GFP expression. Single-locus UBER variants of *B. subtilis* were cloned on the pDG1661 integration vector^70^ (BGSCID# ECE112) after removing the *spoVG-lacZ* region (*ampR*, *cmR*, *spcR*, ColE1 replication origin). For the dual-locus UBER variants of *B. subtilis*, the *T7RNAP* and *tetR* cassettes were cloned on the same modified pDG1661 integration vector, and the *gfp* or the *crtEBI* cassette was cloned on a pDG1661 integration vector further modified to replace the *amyE* homologous arms with *lacA* homologous arms for genomic integration. In all cases, the vector-only control consisted of the modified plasmid backbone circularized without the UBER cassette. Gene cassette organizations are described in Supplementary Table 2, and the relevant transcription signal sequences are listed in Supplementary Tables 3 and 4.

### Growth and fluorescence measurement

For all three bacterial species, growth rates and steady-state fluorescence measurements were recorded using M1000 spectrophotometer (TECAN) and LSR-II Fortessa flow cytometer (BD biosciences), respectively. UBER variants and a control vector-only transformant were inoculated in triplicate into 500 μl LB broth each, in a 96-deep-well plate, and incubated overnight at the appropriate temperature with 200 r.p.m. orbital shaking. Five microlitres of the overnight culture were diluted into 195 μl LB media in a 96-well microtiter plate. The plate was incubated in the M1000 spectrophotometer with high orbital shaking at the appropriate temperature, and its OD_600_ recorded every 10–15 min. The LB media at all stages was supplemented with the appropriate antibiotic/s at the relevant concentrations. Once the cultures reached an OD_600_ of ∼0.20, they were diluted into a new microtiter plate and reincubated, maintaining them within the exponential growth phase. Separately, a sample of each culture was transferred to a new plate containing 200 μl 1 × PBS (NaCl 137 mM, KCl 2.7 mM, Na_2_HPO_4_ 10 mM, KH_2_PO_4_ 1.8 mM, pH 7.4, EMD Millipore# M6506) and 2 mg ml^−1^ kanamycin for flow cytometry measurements. Serial dilutions were carried out one more time. Growth rates were calculated from the exported OD_600_ data. Single-cell fluorescence distributions of samples were measured from the second and the third microtiter plates using the Fortessa flow cytometer. The arithmetic mean of each distribution was taken, and the mean auto-fluorescence of the control was subtracted from each sample.

For the time-course monitoring of fluorescence in *E. coli* UBER PFL variants, cells were streaked on LB agar plates, incubated at 37 °C for ∼16 h, cooled at 4 °C for ∼20 h and then three colonies each were inoculated into 600 μl LB broth in a 96-deep-well plate and incubated at 37 °C with 250 r.p.m. orbital shaking. After 110 min of incubation, 50 μl of fresh media pre-warmed to 37 °C was mixed into the culture, and an equal volume was transferred to a new plate containing 150 μl 1 × PBS and 2 mg ml^−1^ kanamycin end concentration for flow cytometry measurements. Samples were extracted every 45 min.

### Neurosporene extraction and measurement

For measuring neurosporene production, 50 ml bacterial cultures were grown in a shaker incubator (250 r.p.m.) for 24 h at their optimal growth temperature. Cells were pelleted by centrifugation, washed in 1 ml distilled water and dispersed in 1 ml acetone by vortexing. The samples were incubated at 55 °C for 20 min with vortexing at regular intervals, following which they were centrifuged for 5 min, and the supernatant was saved in fresh tubes. OD_470_ of the supernatant was measured using NanoDrop 2000c spectrophotometer and converted to neurosporene in micrograms ( × 3.43 μg per absorbance). The remaining pellet was dried at 60 °C for 48 h to determine dry cell weight. Neurosporene per gram dry cell weight (gDCW) was calculated by normalizing the amount of neurosporene in micrograms by dry cell weight in grams. As negative controls, the 470-nm absorbances of *E. coli*, *B. subtilis* and *P. putida* cell extracts were measured, and subtracted from all neurosporene absorbance measurements.

### Computational modelling

The RBS Calculator v2.0 was employed to calculate the ribosome's binding free energy to bacterial mRNA sequences, and to predict the translation initiation rate of a protein coding sequence. The thermodynamic model uses a five-term Gibbs free energy calculation to quantify the strengths of the molecular interactions between the 30S ribosomal pre-initiation complex and the mRNA region surrounding a start codon^39^. The total Gibbs free energy change is related to the mRNA's translation initiation rate (*r*) according to: *r*=*K*exp(−*β*Δ*G*_total_), where the apparent Boltzmann constant (*β*) has been measured to be about 0.45 mol kcal^−1^ across different bacterial species^5^. The proportionality constant *K* is 2,500 for all predicted translation initiation rates. The error in the prediction is 2.3-fold across a 100,000-fold scale^49^. This software is available at http://salislab.net/software.

The mechanistic model is a system of ordinary differential equations quantifying the rates of production and degradation/dilution for T7 RNAP, TetR and GFP. Four versions of the model were developed to simulate the dynamics of the constitutively expressed T7 RNAP, the expression of T7 RNAP using a PFL, the TetR-repressed expression of T7 RNAP with a PFL, and the regulated expression of both TetR and T7 RNAP using mixed PFL and NFL. The differential equations were numerically integrated over a 14-h period for modelling time-course experiments and over a 60-h period for determining steady-state solutions using the ode45 solver (MATLAB, Mathworks). All differential equations, initial conditions and parameter values are included in the Supplementary Notes.

## Additional information

**How to cite this article:** Kushwaha, M. & Salis, H. M. A portable expression resource for engineering cross-species genetic circuits and pathways. *Nat. Commun*. 6:7832 doi: 10.1038/ncomms8832 (2015).

## Supplementary Material

Supplementary Information Supplementary Figures 1-8, Supplementary Tables 1-4, Supplementary Notes 1-6 and Supplementary References

## References

[b1] Slusarczyk A. L., Lin A. & Weiss R. Foundations for the design and implementation of synthetic genetic circuits. Nat. Rev. Genet. 13, 406–420 (2012). 22596318 10.1038/nrg3227

[b2] Way J. C., Collins J. J., Keasling J. D. & Silver P. A. Integrating biological redesign: where synthetic biology came from and where it needs to go. Cell 157, 151–161 (2014). 24679533 10.1016/j.cell.2014.02.039

[b3] Nielsen A. A., Segall-Shapiro T. H. & Voigt C. A. Advances in genetic circuit design: novel biochemistries, deep part mining, and precision gene expression. Curr. Opin. Chem. Biol. 17, 878–892 (2013). 24268307 10.1016/j.cbpa.2013.10.003

[b4] Daniel R., Rubens J. R., Sarpeshkar R. & Lu T. K. Synthetic analog computation in living cells. Nature 497, 619–623 (2013). 23676681 10.1038/nature12148

[b5] Farasat I. . Efficient search, mapping, and optimization of multi-protein genetic systems in diverse bacteria. Mol. Syst. Biol. 10, 731 (2014). 24952589 10.15252/msb.20134955 PMC4265053

[b6] Kotula J. W. . Programmable bacteria detect and record an environmental signal in the mammalian gut. Proc. Natl Acad. Sci. USA 111, 4838–4843 (2014). 24639514 10.1073/pnas.1321321111 PMC3977281

[b7] Siuti P., Yazbek J. & Lu T. K. Synthetic circuits integrating logic and memory in living cells. Nat. Biotechnol. 31, 448–452 (2013). 23396014 10.1038/nbt.2510

[b8] Nikel P. I., Martínez-García E. & de Lorenzo V. Biotechnological domestication of pseudomonads using synthetic biology. Nat. Rev. Microbiol. 12, 368–379 (2014). 24736795 10.1038/nrmicro3253

[b9] Biggs B. W., De Paepe B., Santos C. N. S., De Mey M. & Ajikumar P. K. Multivariate modular metabolic engineering for pathway and strain optimization. Curr. Opin. Biotechnol. 29, 156–162 (2014). 24927371 10.1016/j.copbio.2014.05.005

[b10] Prindle A. . Rapid and tunable post-translational coupling of genetic circuits. Nature 508, 387–391 (2014). 24717442 10.1038/nature13238 PMC4142690

[b11] Wang B., Barahona M. & Buck M. Engineering modular and tunable genetic amplifiers for scaling transcriptional signals in cascaded gene networks. Nucleic Acids Res. 42, 9484–9492 (2014). 25030903 10.1093/nar/gku593 PMC4132719

[b12] Yang L. . Permanent genetic memory with> 1-byte capacity. Nat. Methods 11, 1261–1266 (2014). 25344638 10.1038/nmeth.3147 PMC4245323

[b13] Cardinale S. & Arkin A. P. Contextualizing context for synthetic biology–identifying causes of failure of synthetic biological systems. Biotechnol. J. 7, 856–866 (2012). 22649052 10.1002/biot.201200085 PMC3440575

[b14] Kittleson J. T., Wu G. C. & Anderson J. C. Successes and failures in modular genetic engineering. Curr. Opin. Chem. Biol. 16, 329–336 (2012). 22818777 10.1016/j.cbpa.2012.06.009

[b15] Nevozhay D., Zal T. & Balázsi G. Transferring a synthetic gene circuit from yeast to mammalian cells. Nat. Commun. 4, 1451 (2013). 23385595 10.1038/ncomms2471 PMC3573884

[b16] Curran K. A. . Design of synthetic yeast promoters via tuning of nucleosome architecture. Nat. Commun. 5, 4002 (2014). 24862902 10.1038/ncomms5002 PMC4064463

[b17] Artsimovitch I., Svetlov V., Anthony L., Burgess R. R. & Landick R. RNA polymerases from *Bacillus subtilis* and *Escherichia coli* differ in recognition of regulatory signals in vitro. J. Bacteriol. 182, 6027–6035 (2000). 11029421 10.1128/jb.182.21.6027-6035.2000 PMC94735

[b18] Meysman P. . Structural properties of prokaryotic promoter regions correlate with functional features. PloS One 9, e88717 (2014). 24516674 10.1371/journal.pone.0088717 PMC3918002

[b19] Greener A., Lehman S. M. & Helinski D. R. Promoters of the broad host range plasmid RK2: analysis of transcription (initiation) in five species of Gram-negative bacteria. Genetics 130, 27–36 (1992). 1732166 10.1093/genetics/130.1.27 PMC1204802

[b20] Leavitt J. M. & Alper H. S. Advances and current limitations in transcript-level control of gene expression. Curr. Opin. Biotechnol. 34, 98–104 (2015). 25559200 10.1016/j.copbio.2014.12.015

[b21] Radeck J. . The Bacillus BioBrick Box: generation and evaluation of essential genetic building blocks for standardized work with *Bacillus subtilis*. J. Biol. Eng. 7, 29 (2013). 24295448 10.1186/1754-1611-7-29 PMC4177231

[b22] Blazeck J., Liu L., Redden H. & Alper H. Tuning gene expression in Yarrowia lipolytica by a hybrid promoter approach. Appl. Environ. Microbiol. 77, 7905–7914 (2011). 21926196 10.1128/AEM.05763-11 PMC3208987

[b23] Elroy-Stein O. & Moss B. Cytoplasmic expression system based on constitutive synthesis of bacteriophage T7 RNA polymerase in mammalian cells. Proc. Natl Acad. Sci. USA 87, 6743–6747 (1990). 2204064 10.1073/pnas.87.17.6743 PMC54613

[b24] Studier F. W. & Moffatt B. A. Use of bacteriophage T7 RNA polymerase to direct selective high-level expression of cloned genes. J. Mol. Biol. 189, 113–130 (1986). 3537305 10.1016/0022-2836(86)90385-2

[b25] Temme K., Hill R., Segall-Shapiro T. H., Moser F. & Voigt C. A. Modular control of multiple pathways using engineered orthogonal T7 polymerases. Nucleic Acids Res. 40, 1–9 (2012). 22743271 10.1093/nar/gks597 PMC3458549

[b26] Segall-Shapiro T. H., Meyer A. J., Ellington A. D., Sontag E. D. & Voigt C. A. A ‘resource allocator' for transcription based on a highly fragmented T7 RNA polymerase. Mol. Syst. Biol. 10, 742 (2014). 25080493 10.15252/msb.20145299 PMC4299498

[b27] Bryksin A. V. & Matsumura I. Rational design of a plasmid origin that replicates efficiently in both gram-positive and gram-negative bacteria. PloS One 5, e13244 (2010). 20949038 10.1371/journal.pone.0013244 PMC2951906

[b28] Towsey M., Timms P., Hogan J. & Mathews S. A. The cross-species prediction of bacterial promoters using a support vector machine. Comput. Biol. Chem. 32, 359–366 (2008). 18703385 10.1016/j.compbiolchem.2008.07.009

[b29] Trumble W. R. . Protein expression from an *Escherichia coli*/*Bacillus subtilis* multifunctional shuttle plasmid with synthetic promoter sequences. Protein. Expr. Purif. 3, 169–177 (1992). 1392613 10.1016/1046-5928(92)90012-l

[b30] Studier F. W. Use of bacteriophage T7 lysozyme to improve an inducible T7 expression system. J. Mol. Biol. 219, 37–44 (1991). 2023259 10.1016/0022-2836(91)90855-z

[b31] Tan C., Marguet P. & You L. Emergent bistability by a growth-modulating positive feedback circuit. Nat. Chem. Biol. 5, 842–848 (2009). 19801994 10.1038/nchembio.218 PMC2908482

[b32] Müller K. M. & Arndt K. M. Standardization in synthetic biology. Methods Mol. Biol. 813, 23–43 (2012). 22083734 10.1007/978-1-61779-412-4_2

[b33] Sørensen H. P. Towards universal systems for recombinant gene expression. Microb. Cell Fact. 9, 27 (2010). 20433754 10.1186/1475-2859-9-27 PMC2876075

[b34] Ehretsmann C. P., Carpousis A. J. & Krisch H. M. Specificity of *Escherichia coli* endoribonuclease RNase E: *in vivo* and *in vitro* analysis of mutants in a bacteriophage T4 mRNA processing site. Genes Dev. 6, 149–159 (1992). 1730408 10.1101/gad.6.1.149

[b35] Lin-Chao S., Wong T. T., McDowall K. J. & Cohen S. N. Effects of nucleotide sequence on the specificity of rne-dependent and RNase E-mediated cleavages of RNA I encoded by the pBR322 plasmid. J. Biol. Chem. 269, 10797–10803 (1994). 7511607

[b36] Mackie G. A. Secondary structure of the mRNA for ribosomal protein S20. Implications for cleavage by ribonuclease E. J. Biol. Chem. 267, 1054–1061 (1992). 1370457

[b37] McDowall K. J., Lin-Chao S. & Cohen S. N. A+U content rather than a particular nucleotide order determines the specificity of RNase E cleavage. J. Biol. Chem. 269, 10790–10796 (1994). 7511606

[b38] Pertzev A. V. & Nicholson A. W. Characterization of RNA sequence determinants and antideterminants of processing reactivity for a minimal substrate of Escherichia coli ribonuclease III. Nucleic Acids Res. 34, 3708–3721 (2006). 16896014 10.1093/nar/gkl459 PMC1540722

[b39] Espah Borujeni A., Channarasappa A. S. & Salis H. M. Translation rate is controlled by coupled trade-offs between site accessibility, selective RNA unfolding and sliding at upstream standby sites. Nucleic Acids Res. 42, 1–14 (2013). 10.1093/nar/gkt1139 PMC3936740 24234441

[b40] Ng C. Y., Farasat I., Maranas C. D. & Salis H. M. Rational design of a synthetic Entner–Doudoroff pathway for improved and controllable NADPH regeneration. Metab. Eng. 29, 86–96 (2015). 25769287 10.1016/j.ymben.2015.03.001

[b41] Dinçbas V., Heurgué-Hamard V., Buckingham R. H., Karimi R. & Ehrenberg M. Shutdown in protein synthesis due to the expression of mini-genes in bacteria. J. Mol. Biol. 291, 745–759 (1999). 10452886 10.1006/jmbi.1999.3028

[b42] Dubendorff J. W. & Studier F. W. Controlling basal expression in an inducible T7 expression system by blocking the target T7 promoter with lac repressor. J. Mol. Biol. 219, 45–59 (1991). 1902522 10.1016/0022-2836(91)90856-2

[b43] Klumpp S., Dong J. & Hwa T. On ribosome load, codon bias and protein abundance. PloS One 7, e48542 (2012). 23144899 10.1371/journal.pone.0048542 PMC3492488

[b44] Brophy J. A. N. & Voigt C. A. Principles of genetic circuit design. Nat. Methods. 11, 508–520 (2014). 24781324 10.1038/nmeth.2926 PMC4230274

[b45] Del Vecchio D. Modularity, context-dependence, and insulation in engineered biological circuits. Trends Biotechnol. 33, 111–119 (2014). 25544476 10.1016/j.tibtech.2014.11.009

[b46] Mali P., Esvelt K. M. & Church G. M. Cas9 as a versatile tool for engineering biology. Nat. Methods 10, 957–963 (2013). 24076990 10.1038/nmeth.2649 PMC4051438

[b47] Gibson D. G. Enzymatic assembly of overlapping DNA fragments. Methods Enzymol. 498, 349–361 (2011). 21601685 10.1016/B978-0-12-385120-8.00015-2 PMC7149801

[b48] Jiang W., Bikard D., Cox D., Zhang F. & Marraffini L. A. RNA-guided editing of bacterial genomes using CRISPR-Cas systems. Nat. Biotechnol. 31, 233–239 (2013). 23360965 10.1038/nbt.2508 PMC3748948

[b49] Salis H. M., Mirsky E. A. & Voigt C. A. Automated design of synthetic ribosome binding sites to control protein expression. Nat. Biotechnol. 27, 946–950 (2009). 19801975 10.1038/nbt.1568 PMC2782888

[b50] Peters J. M., Vangeloff A. D. & Landick R. Bacterial transcription terminators: the RNA 3'-end chronicles. J. Mol. Biol. 412, 793–813 (2011). 21439297 10.1016/j.jmb.2011.03.036 PMC3622210

[b51] Chen Y.-J. . Characterization of 582 natural and synthetic terminators and quantification of their design constraints. Nat. Methods 10, 659–664 (2013). 23727987 10.1038/nmeth.2515

[b52] Na D. . Metabolic engineering of *Escherichia coli* using synthetic small regulatory RNAs. Nat. Biotechnol. 31, 170–174 (2013). 23334451 10.1038/nbt.2461

[b53] Rodrigo G., Landrain T. E. & Jaramillo A. De novo automated design of small RNA circuits for engineering synthetic riboregulation in living cells. Proc. Natl Acad. Sci. USA 109, 15271–15276 (2012). 22949707 10.1073/pnas.1203831109 PMC3458397

[b54] Topp S. . Synthetic riboswitches that induce gene expression in diverse bacterial species. Appl. Environ. Microbiol. 77, 2199–2199 (2011). 10.1128/AEM.01537-10 PMC2988590 20935124

[b55] Lou C., Stanton B., Chen Y.-J., Munsky B. & Voigt C. A. Ribozyme-based insulator parts buffer synthetic circuits from genetic context. Nat. Biotechnol. 30, 1137–1142 (2012). 23034349 10.1038/nbt.2401 PMC3914141

[b56] Pabo C. O. & Nekludova L. Geometric analysis and comparison of protein-DNA interfaces: why is there no simple code for recognition? J. Mol. Biol. 301, 597–624 (2000). 10966773 10.1006/jmbi.2000.3918

[b57] Shis D. L. & Bennett M. R. Library of synthetic transcriptional AND gates built with split T7 RNA polymerase mutants. Proc. Natl Acad. Sci. USA 110, 5028–5033 (2013). 23479654 10.1073/pnas.1220157110 PMC3612686

[b58] Schaerli Y., Gili M. & Isalan M. A split intein T7 RNA polymerase for transcriptional AND-logic. Nucleic Acids Res. 42, 12322–12328 (2014). 25262348 10.1093/nar/gku884 PMC4231753

[b59] Meyer A. J., Ellefson J. W. & Ellington A. D. Directed evolution of a panel of orthogonal T7 RNA polymerase variants for *in vivo* or *in vitro* synthetic circuitry. ACS Synth. Biol. (2014). 10.1021/sb500299c 25279711

[b60] Siezen R. J. & van Hylckama Vlieg J. Genomic diversity and versatility of Lactobacillus plantarum, a natural metabolic engineer. Microb. Cell Fact. 10, S3 (2011). 21995294 10.1186/1475-2859-10-S1-S3 PMC3271238

[b61] Tsuji A., Okada S., Hols P. & Satoh E. Metabolic engineering of< i> Lactobacillus plantarum</i> for succinic acid production through activation of the reductive branch of the tricarboxylic acid cycle. Enzyme Microb. Technol. 53, 97–103 (2013). 23769309 10.1016/j.enzmictec.2013.04.008

[b62] Kang T. S., Korber D. R. & Tanaka T. Metabolic engineering of a glycerol-oxidative pathway in Lactobacillus panis PM1 for utilization of bioethanol thin stillage: potential to produce platform chemicals from glycerol. Appl. Environ. Microbiol. 80, 7631–7639 (2014). 25281374 10.1128/AEM.01454-14 PMC4249216

[b63] Wu M.-C., Law B., Wilkinson B. & Micklefield J. Bioengineering natural product biosynthetic pathways for therapeutic applications. Curr. Opin. Biotechnol. 23, 931–940 (2012). 22487048 10.1016/j.copbio.2012.03.008

[b64] Lütke-Eversloh T. Application of new metabolic engineering tools for *Clostridium acetobutylicum*. Appl. Microbiol. Biotechnol. 98, 5823–5837 (2014). 24816621 10.1007/s00253-014-5785-5

[b65] Lee J. . Metabolic engineering of *Clostridium acetobutylicum* ATCC 824 for isopropanol-butanol-ethanol fermentation. Appl. Environ. Microbiol. 78, 1416–1423 (2012). 22210214 10.1128/AEM.06382-11 PMC3294493

[b66] Smanski M. J. . Functional optimization of gene clusters by combinatorial design and assembly. Nat. Biotechnol. 32, 1241–1249 (2014). 25419741 10.1038/nbt.3063

[b67] Bron S. in Molecular Biological Methods for Bacillus eds Harwood Colin R., Cutting Simon M. 75–174John Wiley & Son Ltd (1990).

[b68] Merksamer P. I., Trusina A. & Papa F. R. Real-time redox measurements during endoplasmic reticulum stress reveal interlinked protein folding functions. Cell 135, 933–947 (2008). 19026441 10.1016/j.cell.2008.10.011 PMC2739138

[b69] Karsi A. & Lawrence M. L. Broad host range fluorescence and bioluminescence expression vectors for Gram-negative bacteria. Plasmid 57, 286–295 (2007). 17207855 10.1016/j.plasmid.2006.11.002

[b70] Guérout-Fleury A. M., Frandsen N. & Stragier P. Plasmids for ectopic integration in Bacillus subtilis. Gene 180, 57–61 (1996). 8973347 10.1016/s0378-1119(96)00404-0

