# Supplementary material for: A portable expression resource for engineering cross-species genetic circuits and pathways
Source: Nat Commun. 2015 Jul 17;6:7832. doi: 10.1038/ncomms8832 (PMC4518296; doi:10.1038/ncomms8832)
Supplement: Supplementary Information — Supplementary Figures 1-8, Supplementary Tables 1-4, Supplementary Notes 1-6 and Supplementary References [file ncomms8832-s1.pdf]

CCATTATTATCATGACATTAACCTATAAAAATAGGCGTATCACGAGGC**C**[1]CTTTCGTCTTCAAGAATTGATCT
G[1]TCGACAGGAACTGGACAGCGTGTCGAAAAAGCTCGACAGGAACTGGACAGCGTGTCGAAAAAGCTCG
ACAGGAACTGGACAGCGTGTCGAAAAAGCTCGACAGGAACTGGACAGCGTGTCGAAAAAGCTCGAGCAG
ATCCGCCAGGCGTGTATATATAGCGTGGATGGCCAGGCAACTTTAGTGCTGACACATACAGGCATATATA
TATGTGTGCGACGACACATGATCATATGGCATGCATGTGCTCTGTATGTATATAAAACTCTTG**T**[2]TTTCTTCT
TTTCTCTAAATATTCTTTCCTT**A**[2]TACATTAGGTC**C**[3]TTTGTAGCATAAATTACTATACTTCTATAGACACGCA
AACACAAATACACACACTAAATTAATAG**G**[4]ATCC

Supplementary Figure 1 | The 456 nt Priming pr sequence contains some promoter-like elements.

The above sequence was analysed using a Neural Network Promoter Prediction algorithm (NNPP[1],http://www.fruitfly.org/seq_tools/promoter.html), as well as an automated genome annotation tool (BPROM[2], http://linux1.softberry.com/berry.phtml). The four positions marked in red are the putative transcription start sites predicted by NNPP, with scores of 0.83, 0.89, 0.97 and 0.87 in order. The two positions marked in green are the putative transcription start sites predicted by BPROM, with scores of 1.16 and 5.23 in order.

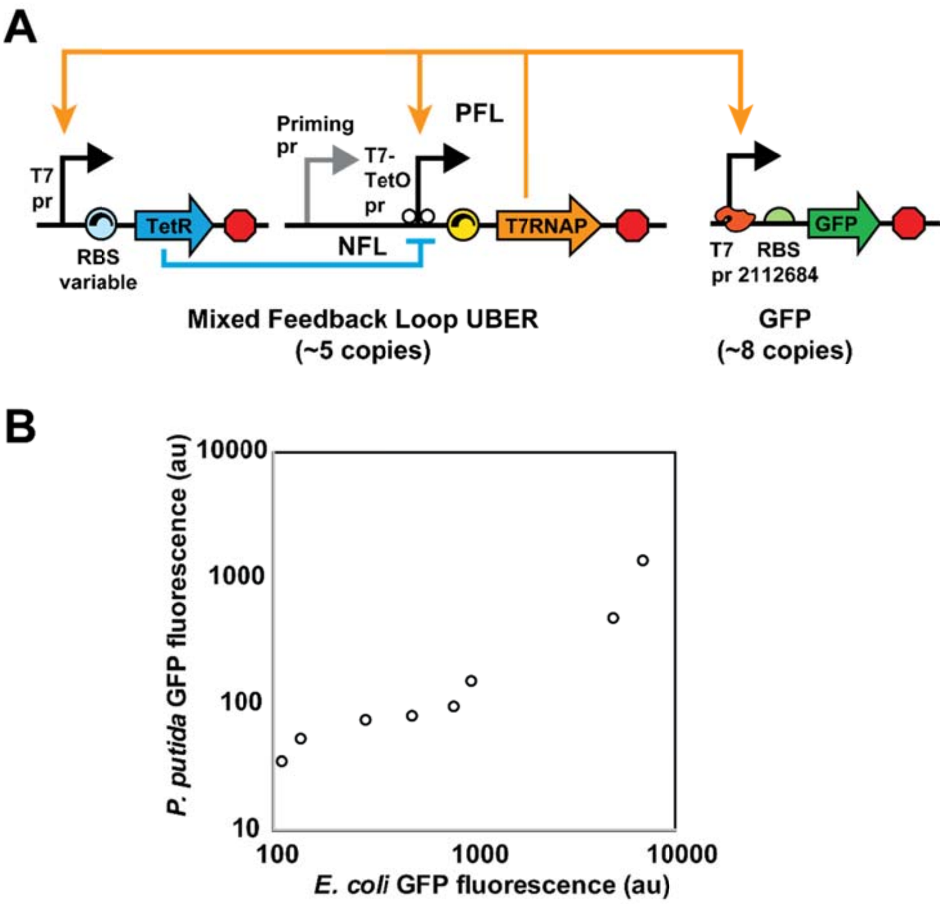

Supplementary Figure 2 | Relative expression from UBER variants is similar across species.

(A) The figure shows the genetic implementation of a dual-plasmid Mixed Feedback Loop (MFL) UBER version.

(B) The figure plots GFP fluorescence (au) of eight MFL UBER variants in *E. coli* against their GFP fluorescence (au) in *P. putida*. *E. coli* were cultured at 37 °C, while *P. putida* were cultured at 30 °C.

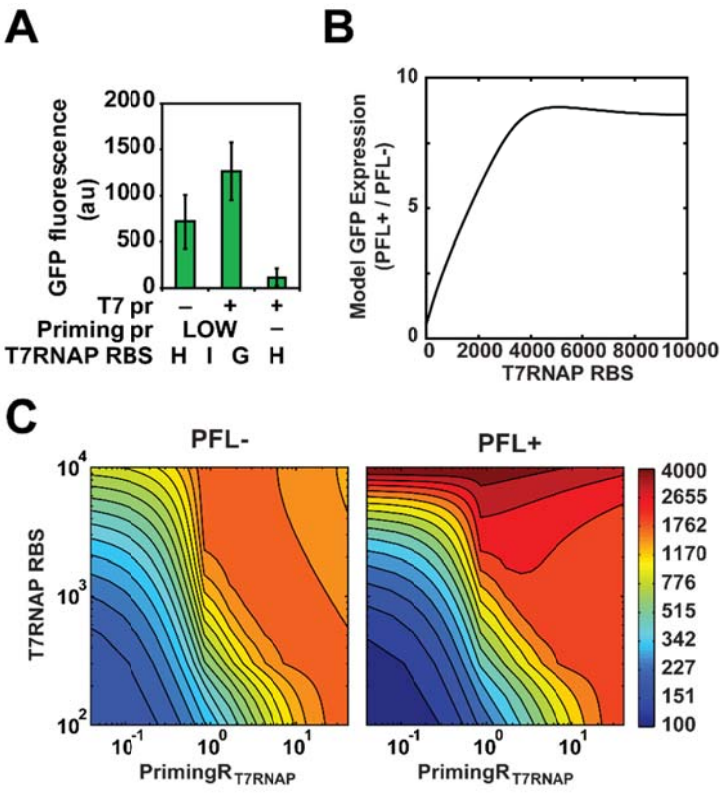

Supplementary Figure 3 | Both Basal transcription and T7RNAP translation rate modulate Positive Feedback Loop output.

(A) The first two bars show GFP expression of steady state cells in the open loop (PFL-) and the closed-loop (PFL+) variants of the low-Priming/ high-T7 RNAP RBS UBER version (same as the two rightmost bars in Figure 2B, top graph). The third bar shows the reduction in expression when the Priming pr is deleted, reducing the basal transcription to a minimal despite the PFL.

(B) The graph shows the ratio of GFP steady state model solutions for the closed-loop (PFL+) versus the open-loop (PFL-) UBER system across a range of T7 RNAP translation rates.

(C) The two contour plots show the steady state solutions for GFP for a range of basal T7RNAP transcription and RBS translation rates in PFL- and the PFL+ variants.

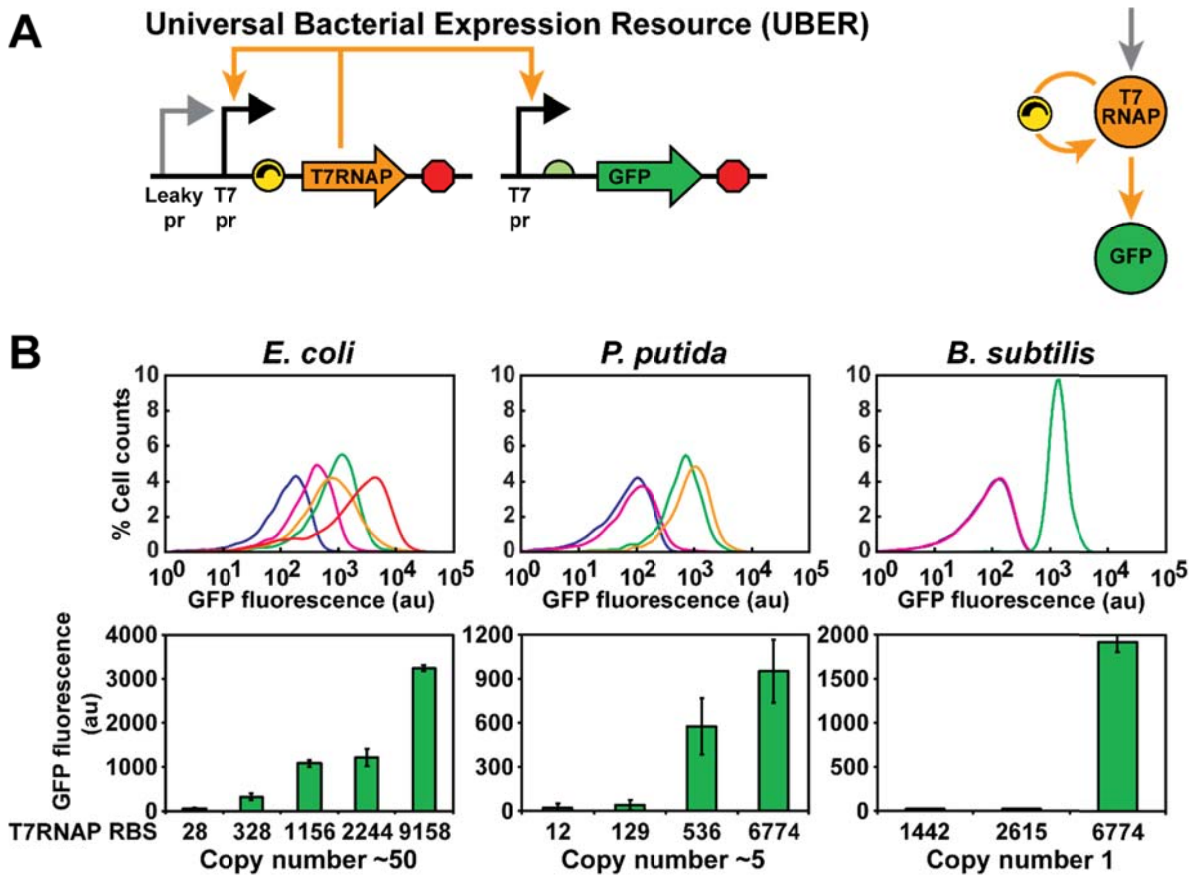

Supplementary Figure 4 | Positive Feedback Loop enables tunability and cross-species portability.

(A) The figure shows the genetic implementation of the closed-loop Positive Feedback Loop (PFL+) UBER version.

(B) The top panel shows the population distributions of GFP fluorescence for different closed-loop (PFL+ UBER variants in three bacterial species–*E. coli*, *P. putida* and *B. subtilis*– with tunable output expression. The bottom panel shows the mean fluorescence values for the same clones.

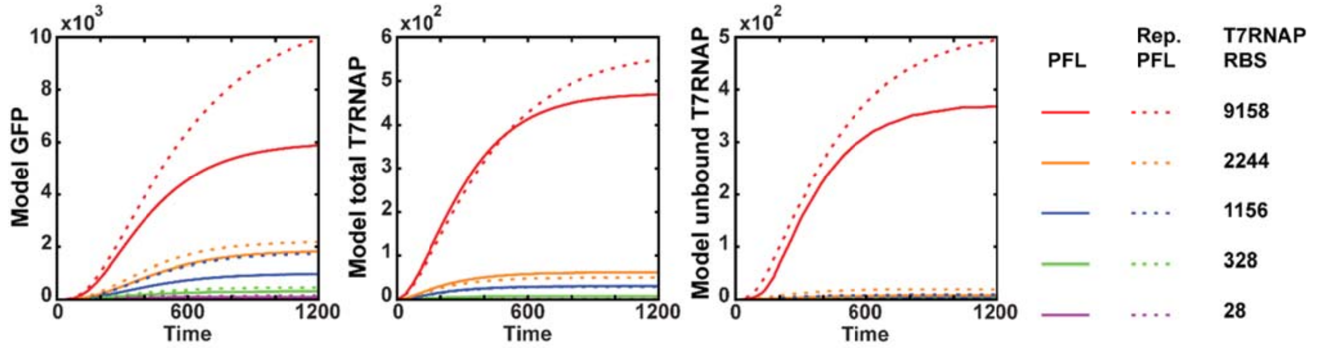

Supplementary Figure 5 | Constitutive TetR increases GFP expression in the Repressed Feedback Loop UBER system, despite lower total cellular T7RNAP.

The three plots show the time course model simulations for total GFP, total T7RNAP, and the unbound T7RNAP in the cell- for both the PFL and the Rep. PFL UBER versions. Rep. PFL achieves higher GFP expression due to differential partitioning. However, there is also higher unbound T7RNAP in the Rep. PFL cells.

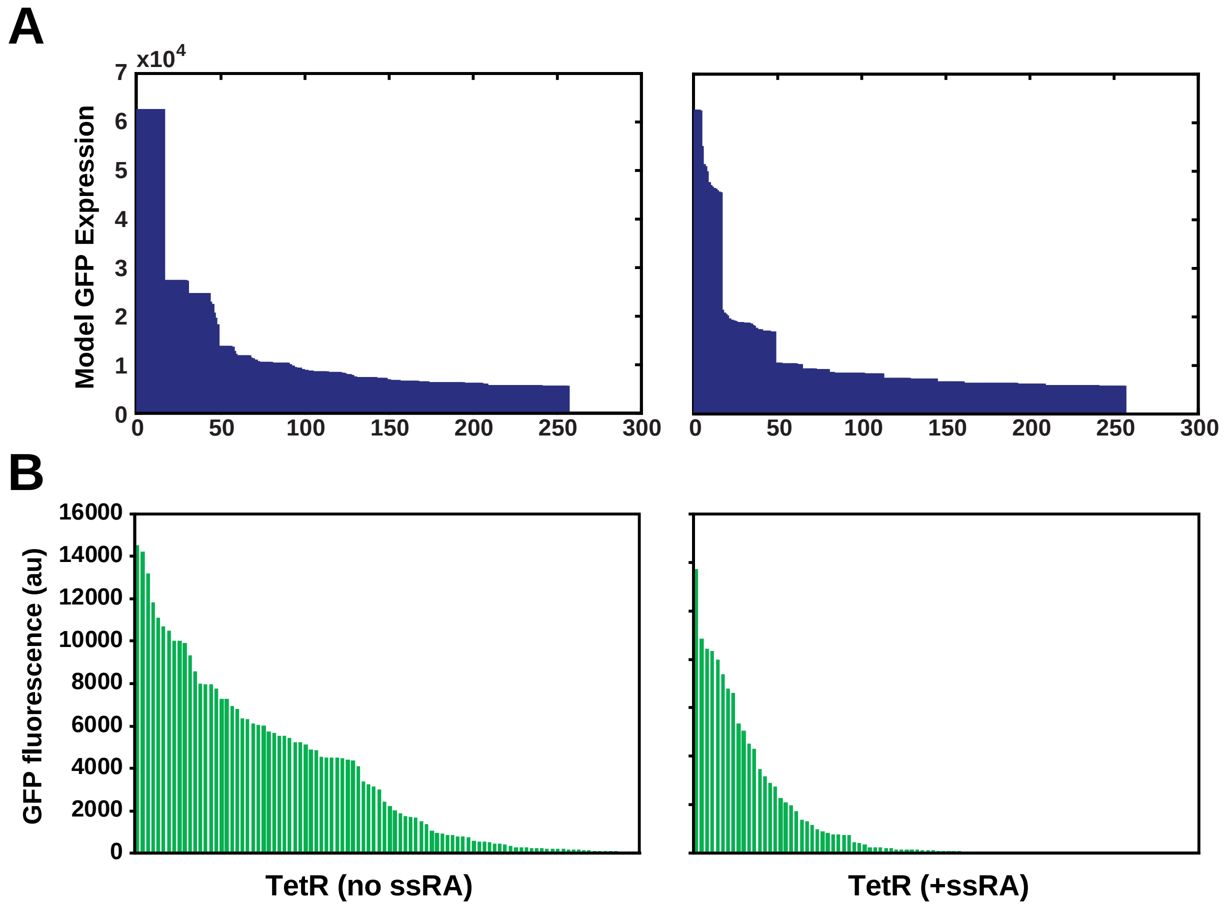

Supplementary Figure 6 | Decreasing TetR half-life results in lower GFP expression in the Mixed Feedback Loop UBER system.

(A) The bar graphs show the model GFP output for a combinatorial library of 16x16 RBSes of T7RNAP and TetR expression in the Mixed Feedback Loop UBER system, sorted in descending order. Model output from TetR (without ssRA tag) library is on the left, while that from TetR+ssRA tag library is on the right.

(B) The bar graphs show steady state GFP expression from 96 members of a combinatorial library of 16x16 RBSes of T7RNAP and TetR expression in the Mixed Feedback Loop UBER system, sorted in descending order. Model output from TetR (without ssRA tag) library is on the left, while that from TetR+ssRA tag library is on the right.

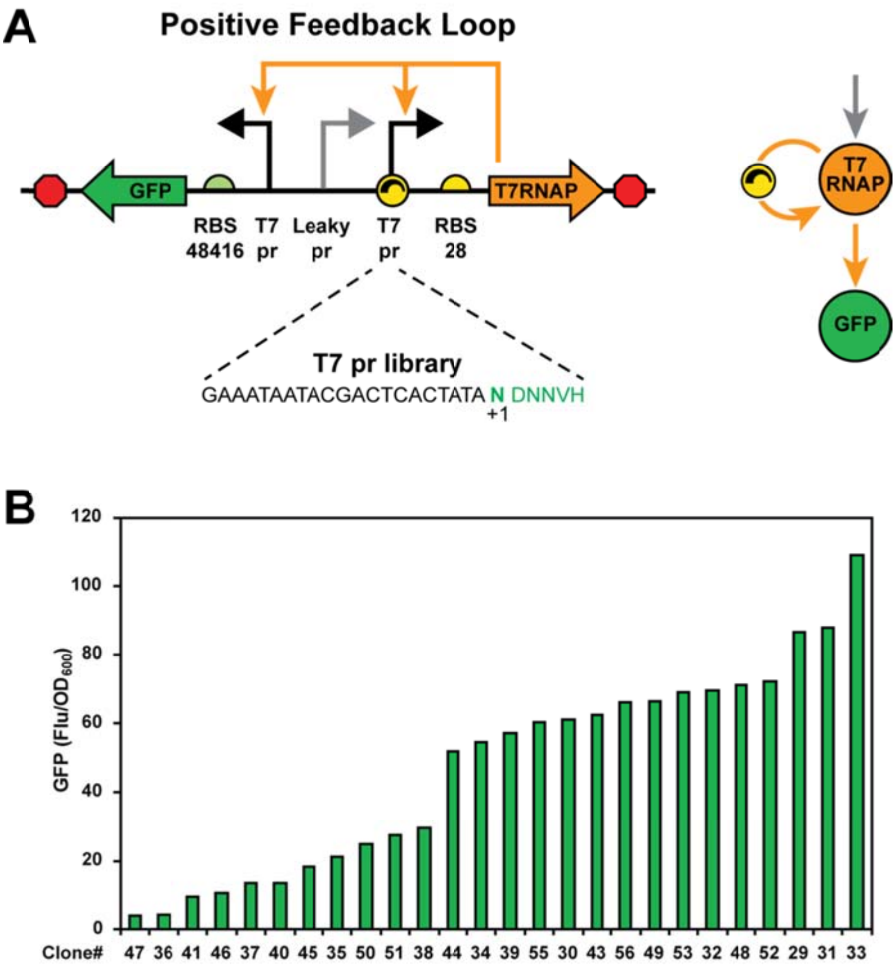

Supplementary Figure 7 | A toolbox of T7 promoters for controlling the output module's expression levels

(A) A degenerate T7 promoter sequence that varies the T7 RNAP's binding affinity was inserted into the PFL(+) variant of the UBER system using a strategy similar to a previous study[4].

(B) 33 individual variants were transformed into *E. coli* DH10B and characterized in LB media at 37°C over an 8-hour culture. GFP fluorescence was measured by spectrophotometry. The measured output module expression levels varied by 24-fold.

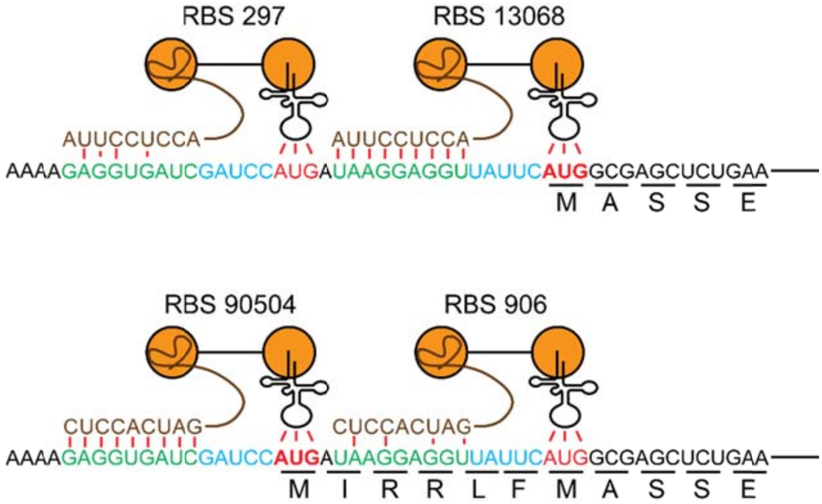

Supplementary Figure 8 | Staggered in-frame RBS design for bacteria with divergent anti-SD sequences.

A staggered in-frame RBS design facilitates high translation across bacteria with highly divergent anti-Shine Dalgarno (aSD) sequences. The top panel shows an organism with 5’-ACCUCCUUA-3’ aSD sequence (*E. coli*, for example) that preferentially initiates translation from the second in-frame start codon. For the same DNA construct, organism in the bottom panel with a divergent 5’-GAUCACCUC-3’ aSD preferentially initiates translation from the first in-frame start codon. The RBS strengths for both the organisms can be calculated using the RBS calculator.

Supplementary Table 1

Optimized coding sequences	
**Protein**	**Coding Sequence (5’->3’)**
T7RNAP<br>(2697 nt)	ATGTCTACCACCATGGGAATTCAACCTCCTAAAAAGAAACGTAAAGTTAATACT<br>ATTAATATTGCTAAAAATGACTTCTCAGATATTGAATTAGCAGCCATTCCATTTA<br>ATACATTAGCAGATCACTATGGTGAACGTTTAGCACGTGAACAGTTAGCATTAG<br>AACATGAATCATATGAAATGGGTGAAGCACGTTTTCGTAAGATGTTCGAGCGT<br>CAGTTAAAAGCAGGTGAAGTTGCAGATAATGCAGCAGCCAAACCTTTAATTACT<br>ACATTATTACCTAAAATGATTGCTCGTATTAACGATTGGTTTGAAGAGGTTAAA<br>GCAAAGCGTGGTAAACGTCCTACAGCATTTCAGTTCTTACAAGAAATCAAACCT<br>GAAGCAGTTGCATATATTACTATTAAAACAACATTAGCATGTTTAACATCAGCA<br>GATAATACAACAGTTCAAGCAGTTGCATCAGCAATTGGTCGTGCAATTGAAGAT<br>GAAGCACGTTTTGGTCGTATTCGTGATTTAGAAGCCAAACATTTTAAAAAAAAT<br>GTTGAAGAACAGTTAAACAAACGTGTTGGTCATGTTTATAAAAAAGCATTTATG<br>CAGGTTGTTGAAGCAGATATGTTATCAAAAGGTTTATTAGGTGGTGAAGCATG<br>GTCATCATGGCATAAAGAAGATTCAATTCATGTTGGTGTTCGTTGTATTGAAAT<br>GTTAATTGAATCAACAGGTATGGTTTCATTACATCGTCAGAATGCAGGTGTTGT<br>TGGTCAAGATTCAGAAACAATTGAATTAGCACCTGAATATGCAGAAGCAATTGC<br>AACACGTGCAGGTGCATTAGCAGGTATTTCACCAATGTTTCAGCCTTGTGTTGT<br>TCCTCCTAAACCTTGGACAGGTATTACAGGTGGTGGTTATTGGGCAAATGGTC<br>GTCGTCCTTTAGCATTAGTTCGTACACATTCAAAAAAAGCATTAATGCGTTATG<br>AAGATGTTTACATGCCTGAAGTTTATAAAGCCATTAATATTGCACAGAATACTG<br>CATGGAAAATCAACAAGAAAGTTTTAGCAGTTGCAAATGTTATTACTAAATGGA<br>AACATTGTCCTGTTGAAGATATTCCTGCAATTGAACGTGAAGAATTACCAATGA<br>AACCTGAAGATATTGATATGAATCCTGAAGCATTAACAGCATGGAAACGTGCA<br>GCAGCAGCCGTTTATCGTAAAGATAAAGCACGTAAATCACGTCGTATTTCATTA<br>GAGTTCATGTTAGAGCAAGCAAACAAGTTCGCAAATCATAAAGCCATTTGGTTT<br>CCTTATAATATGGATTGGCGTGGTCGTGTTTATGCAGTTTCAATGTTTAATCCT<br>CAAGGTAATGATATGACCAAAGGTTTATTAACCTTAGCTAAAGGTAAACCTATT<br>GGTAAAGAAGGTTATTATTGGTTAAAAATCCATGGTGCAAATTGTGCAGGTGTT<br>GATAAAGTTCCTTTTCCAGAACGTATTAAGTTCATTGAAGAAAATCATGAAAATA<br>TTATGGCATGTGCTAAATCACCATTAGAAAATACATGGTGGGCAGAACAAGATT<br>CACCTTTTTGTTTTTTAGCCTTTTGTTTTGAATATGCAGGTGTTCAACATCATGG<br>TTTATCATATAATTGTTCATTACCATTAGCATTTGATGGTTCATGTTCAGGTATT<br>CAGCATTTTTCAGCAATGTTACGTGATGAAGTTGGTGGTCGTGCAGTTAACTTA<br>TTACCTTCAGAAACAGTTCAAGATATCTATGGTATTGTTGCTAAAAAAGTTAATG<br>AAATCTTACAGGCAGATGCCATTAATGGTACAGATAATGAAGTTGTTACAGTTA<br>CAGATGAAAATACAGGTGAAATATCAGAAAAAGTTAAATTAGGTACCAAAGCAT<br>TAGCAGGTCAATGGTTAGCATATGGTGTTACACGTTCAGTTACTAAGCGTTCAG<br>TTATGACATTAGCATATGGTTCAAAAGAGTTCGGTTTTCGTCAACAGGTTTTAG<br>AAGATACCATTCAACCTGCAATTGATTCAGGTAAAGGTTTAATGTTTACTCAAC<br>CTAATCAAGCAGCAGGTTATATGGCCAAATTAATATGGGAATCAGTTTCAGTTA<br>CAGTTGTTGCAGCAGTTGAAGCAATGAATTGGTTAAAATCAGCAGCCAAATTAT<br>TAGCAGCAGAAGTTAAAGATAAAAAAACAGGTGAAATCTTACGTAAGCGTTGTG<br>CAGTTCATTGGGTTACACCTGATGGTTTTCCTGTTTGGCAAGAATATAAAAAAC<br>CTATTCAAACACGTTTAAACTTAATGTTTTTAGGTCAGTTCCGTTTACAACCTAC<br>TATTAATACAAACAAAGATTCAGAAATTGATGCACATAAACAAGAATCAGGTATT<br>GCACCTAACTTCGTTCATTCACAAGATGGTTCACATTTACGTAAAACAGTTGTT<br>TGGGCACATGAAAAATATGGTATTGAATCATTTGCATTAATTCACGATTCATTTG<br>GTACCATTCCAGCAGATGCAGCAAACTTATTCAAAGCAGTTCGTGAAACAATG<br>GTTGATACATATGAATCATGTGATGTTTTAGCAGACTTCTATGATCAGTTCGCA

	GATCAGTTACATGAATCACAGTTAGATAAAATGCCTGCATTACCTGCCAAAGGT<br>AACTTAAACTTACGTGATATTTTAGAATCAGACTTCGCATTTGCCTAA
GFP<br>(744 nt)	ATGTCTACCACCATGGGCGACGTCATGGTTTCAAAGGGTGAAGAATTATTTAC<br>AGGTGTTGTTCCTATTTTAGTTGAATTAGATGGTGATGTTAATGGTCATAAGTTC<br>TCAGTTTCAGGTGAAGGTGAAGGTGATGCAACATATGGTAAGTTAACATTAAAG<br>TTCATTTGTACAACAGGTAAATTACCTGTTCCTTGGCCTACATTAGTTACAACAT<br>TAACATATGGTGTTCAATGTTTTTCACGTTATCCTGATCATATGAAACAACATGA<br>CTTCTTTAAATCAGCAATGCCAGAAGGTTACGTTCAAGAACGTACCATCTTTTT<br>TAAAGATGATGGTAACTATAAAACACGTGCAGAAGTTAAGTTCGAAGGTGATAC<br>ATTAGTTAATCGTATTGAATTAAAAGGTATTGACTTCAAAGAAGATGGTAATATT<br>TTAGGTCATAAATTAGAATATAACTATAATTCACATAATGTTTATATTATGGCAG<br>ATAAACAGAAAAATGGTATTAAAGTTAACTTCAAGATACGTCATAATATTGAAGA<br>TGGTTCAGTTCAGTTAGCAGATCATTATCAACAGAATACTCCTATTGGTGATGG<br>TCCTGTTTTATTACCTGATAATCATTACTTATCAACCCAATCAGCATTATCAAAA<br>GATCCTAATGAAAAACGTGATCATATGGTTTTATTAGAGTTCGTTACAGCAGCA<br>GGTATTACATTAGGTATGGATGAATTATATAAATGA
TetR (+ssRA)<br>(702 nt)	ATGTCTACCACCATGGAGATCTATCCTCCTAAAAAAAAACGTAAAGTTTCACGT<br>TTAGATAAATCAAAAGTTATTAACTCAGCATTAGAATTATTAAATGAAGTTGGTA<br>TCGAAGGTTTAACTACACGTAAATTAGCACAGAAATTAGGTGTTGAACAACCTA<br>CATTATATTGGCATGTTAAAAACAAACGTGCATTATTAGATGCATTAGCAATCG<br>AAATGTTAGATAGACATCATACACATTTTTGTCCATTAGAAGGAGAATCATGGC<br>AAGACTTCTTACGTAACAATGCCAAATCATTTCGTTGTGCATTATTATCACATAG<br>AGATGGTGCCAAAGTTCACTTAGGTACACGTCCTACAGAAAAACAATATGAAAC<br>ATTAGAAAATCAGTTAGCATTCTTATGTCAACAAGGTTTTTCATTAGAAAACGCA<br>TTATATGCATTATCAGCAGTTGGTCATTTTACCTTAGGTTGTGTTTTAGAAGATC<br>AAGAACATCAAGTTGCCAAGGAGGAAAGAGAAACACCTACAACAGATTCAATG<br>CCTCCTTTATTACGTCAAGCAATTGAATTATTTGATCATCAAGGTGCAGAACCT<br>GCATTTTTATTTGGTTTAGAATTAATCATTTGCGGTTTGGAGAAACAGTTAAAAT<br>GTGAATCAGGTTCA-**GCAGCAAATGATGAAAACTATGCATTAGTTGCATAA**
TetR (-ssRA)<br>(669 nt)	ATGTCTACCACCATGGAGATCTATCCTCCTAAAAAAAAACGTAAAGTTTCACGT<br>TTAGATAAATCAAAAGTTATTAACTCAGCATTAGAATTATTAAATGAAGTTGGTA<br>TCGAAGGTTTAACTACACGTAAATTAGCACAGAAATTAGGTGTTGAACAACCTA<br>CATTATATTGGCATGTTAAAAACAAACGTGCATTATTAGATGCATTAGCAATCG<br>AAATGTTAGATAGACATCATACACATTTTTGTCCATTAGAAGGAGAATCATGGC<br>AAGACTTCTTACGTAACAATGCCAAATCATTTCGTTGTGCATTATTATCACATAG<br>AGATGGTGCCAAAGTTCACTTAGGTACACGTCCTACAGAAAAACAATATGAAAC<br>ATTAGAAAATCAGTTAGCATTCTTATGTCAACAAGGTTTTTCATTAGAAAACGCA<br>TTATATGCATTATCAGCAGTTGGTCATTTTACCTTAGGTTGTGTTTTAGAAGATC<br>AAGAACATCAAGTTGCCAAGGAGGAAAGAGAAACACCTACAACAGATTCAATG<br>CCTCCTTTATTACGTCAAGCAATTGAATTATTTGATCATCAAGGTGCAGAACCT<br>GCATTTTTATTTGGTTTAGAATTAATCATTTGCGGTTTGGAGAAACAGTTAAAAT<br>GTGAATCAGGTTCA-**TAA**

Supplementary Table 2

Gene Cassette Organization								
**UBER version**	**Relevant Figures***	**Cassette**	**5'->3' organization**					
			**Transcription Initiation Signals**		**Translation Signals**	**Coding Sequence (CDS)**	**Transcription Termination Signals**	
PFL- LOW Priming	2, 3, S3	T7RNAP	LOW Priming pr	-	RBS	optimized T7RNAP	BBa_B0015	SBa_000587
PFL- HIGH Priming	2	T7RNAP	HIGH Priming pr	-	RBS	optimized T7RNAP	BBa_B0015	SBa_000587
PFL+ (*E. coli* & *P. putida*), R-PFL & MFL	1, 2, 3, 4, 5, S2, S3, S4, S6	T7RNAP	LOW Priming pr	T7 pr (TetO)	RBS	optimized T7RNAP	BBa_B0015	SBa_000587
PFL+ (*B. subtilis*)	S4	T7RNAP	LOW Priming pr	T7 pr	RBS	optimized T7RNAP	BBa_B0015	SBa_000587
PFL+	S7	T7RNAP	LOW Priming pr	deg T7 pr	RBS	optimized T7RNAP	BBa_B0015	SBa_000587
PFL-, PFL+, R-PFL & MFL	1, 2, 3, 4, 5, S2, S3, S4, S6, S7	GFP	-	T7 pr	RBS	optimized GFP	-	BBa_Z0272
R-PFL	4	TetR	-	J23102 pr	RBS	optimized TetR(+ssRA)	BBa_B1002	SBa_000451
MFL	1, 5, S6	TetR	-	T7 pr	RBS	optimized TetR(-/+ssRA)	BBa_B1002	SBa_000451
*Prefix 'S' indicates Supplementary Figure.								

Supplementary Table 3

Transcription Initiation Signals	
**Promoter**	**Sequence**
LOW Priming pr	CCATTATTATCATGACATTAACCTATAAAAATAGGCGTATCACGAGGCCCTTTCGTCTT<br>CAAGAATTGATCTGTCGACAGGAACTGGACAGCGTGTCGAAAAAGCTCGACAGGAACT<br>GGACAGCGTGTCGAAAAAGCTCGACAGGAACTGGACAGCGTGTCGAAAAAGCTCGAC<br>AGGAACTGGACAGCGTGTCGAAAAAGCTCGAGCAGATCCGCCAGGCGTGTATATATA<br>GCGTGGATGGCCAGGCAACTTTAGTGCTGACACATACAGGCATATATATATGTGTGCG<br>ACGACACATGATCATATGGCATGCATGTGCTCTGTATGTATATAAAACTCTTGTTTTCTT<br>CTTTTCTCTAAATATTCTTTCCTTATACATTAGGTCCTTTGTAGCATAAATTACTATACTT<br>CTATAGACACGCAAACACAAATACACACACTAAATTAATA-**GGATCC**
HIGH Priming pr	CCATTATTATCATGACATTAACCTATAAAAATAGGCGTATCACGAGGCCCTTTCGTCTT<br>CAAGAATTGATCTGTCGACAGGAACTGGACAGCGTGTCGAAAAAGCTCGACAGGAACT<br>GGACAGCGTGTCGAAAAAGCTCGACAGGAACTGGACAGCGTGTCGAAAAAGCTCGAC<br>AGGAACTGGACAGCGTGTCGAAAAAGCTCGAGCAGATCCGCCAGGCGTGTATATATA<br>GCGTGGATGGCCAGGCAACTTTAGTGCTGACACATACAGGCATATATATATGTGTGCG<br>ACGACACATGATCATATGGCATGCATGTGCTCTGTATGTATATAAAACTCTTGTTTTCTT<br>CTTTTCTCTAAATATTCTTTCCTTATACATTAGGTCCTTTGTAGCATAAATTACTATACTT<br>CTATAGACACGCAAACACAAATACACACACTAAATTAATA-**ACTAGT-**<br>**GTTGACGGCTAGCTCAGTCCTAGGTACAGTGCTAGC**
T7 pr (TetO)	TCTATCATTGATAGG-TAT-AAATTAATACGACTCACTATAGG-GAGA-<br>CCTATCAGTGATAGA
T7 pr	AAATTAATACGACTCACTATAGG
deg T7 pr	GAAATAATACGACTCACTATA-**NDNNVH**
J23102 pr	TTGACAGCTAGCTCAGTCCTAGGTACTGTGCTAGC

Supplementary Table 4

Transcription Termination Signals	
**Intrinsic Terminator**	**Sequence**
BBa_B0015	CCAGGCATCAAATAAAACGAAAGGCTCAGTCGAAAGACTGGGCCTTTCGTTTT ATCTGTTGTTTGTCGGTGAACGCTCTCTACTAGAGTCACACTGGCTCACCTTC GGGTGGGCCTTTCTGCGTTTATA
BBa_B1002	CGCAAAAAACCCCGCTTCGGCGGGGTTTTTTCGC
**T7 terminator**	**Sequence**
SBa_000587	TACTCGAACCCCTAGCCCGCTCTTATCGGGCGGCTAGGGGTTTTTTGT
BBa_Z0272	AACCCCTTGGGGCCTCTAAACGGGTCTTGAGGGGTTTTTTG
SBa_000451	TACATATCGGGGGGGTAGGGGTTTTTTGT

Supplementary Note 1

Open Loop (PFL¯)

ODEs

$$\frac{d[mRNA_{T7RNAP}]}{dt} = CopyN \,.\, primingR_{T7RNAP} - \delta_{mRNA} \,.\, [mRNA_{T7RNAP}] \qquad (1)$$

$$\frac{d[T7RNAP]}{dt} = PR_{T7RNAP} \,.\, [mRNA_{T7RNAP}] - \delta_{T7RNAP} \,.\, [T7RNAP] \qquad (2)$$

$$\frac{d[mRNA_{GFP}]}{dt} = CopyN \,.\, (leakyR_{GFP} + R_{T7prGFP}) - \delta_{mRNA} \,.\, [mRNA_{GFP}] \qquad (3)$$

$$\frac{d[GFP]}{dt} = PR_{GFP} . [mRNA_{GFP}] - \delta_{GFP} \,.\, [GFP] \qquad (4)$$

Reaction Mechanism

$$\boldsymbol{T7RNAP_{free} + T7prGFP_{free} \underset{k_b}{\overset{k_f}{\leftrightarrows}} PIC_{GFP} \xrightarrow{k_{init}} EC_{GFP} \xrightarrow{k_{elong}} T7RNAP_{free} + T7prGFP_{free} + mRNA_{GFP}}$$

$$\frac{d[PIC_{GFP}]}{dt} = k_f \,.\, [T7RNAP_{free}] . [T7prGFP_{free}] - \mathrm{k}_b \,.\, [PIC_{GFP}] - \mathrm{k}_{init} \,.\, [PIC_{GFP}] \qquad (5)$$

$$\frac{d[EC_{GFP}]}{dt} = k_{init} \,.\, [PIC_{GFP}] - \frac{k_{elong}}{L_{GFP}} \,.\, [EC_{GFP}] \qquad (6)$$

$$\frac{d[mRNA_{GFP}]}{dt} = k_{init} \,.\, [PIC_{GFP}] \qquad (7)$$

Mole Balances

$$[T7prGFP_{total}] = [T7prGFP_{free}] + [PIC_{GFP}] \qquad (8)$$

$$[T7RNAP_{total}] = [T7RNAP_{free}] + [PIC_{GFP}] + [EC_{GFP}] \qquad (9)$$

*Symbol descriptions and parameter values are listed in Supplementary Note 5.

Supplementary Note 2

<u>Positive Feedback Loop (PFL⁺)</u>

ODEs

$$\frac{d[mRNA_{T7RNAP}]}{dt} = CopyN\ .\ (primingR_{T7RNAP} + R_{T7prT7RNAP}) - \delta_{mRNA}\ .\ [mRNA_{T7RNAP}] \quad (1)$$

$$\frac{d[T7RNAP]}{dt} = PR_{T7RNAP}\ .\ [mRNA_{T7RNAP}] - \delta_{T7RNAP}\ .\ [T7RNAP] \quad (2)$$

$$\frac{d[mRNA_{GFP}]}{dt} = CopyN\ .\ (leakyR_{GFP} + R_{T7prGFP}) - \delta_{mRNA}\ .\ [mRNA_{GFP}] \quad (3)$$

$$\frac{d[GFP]}{dt} = PR_{GFP}.\ [mRNA_{GFP}] - \delta_{GFP}\ .\ [GFP] \quad (4)$$

Reaction Mechanism

$$\boldsymbol{T7RNAP_{free} + T7prGFP_{free} \underset{k_b}{\overset{k_f}{\leftrightarrows}} PIC_{GFP} \xrightarrow{k_{init}} EC_{GFP} \xrightarrow{k_{elong}} T7RNAP_{free} + T7prGFP_{free} + mRNA_{GFP}}$$

$$\boldsymbol{T7RNAP_{free} + T7prT7RNAP_{free} \underset{k_b}{\overset{k_f}{\leftrightarrows}} PIC_{T7RNAP} \xrightarrow{k_{init}} EC_{T7RNAP} \xrightarrow{k_{elong}} T7RNAP_{free} + T7prT7RNAP_{free} + mRNA_{T7RNAP}}$$

$$\frac{d[PIC_{GFP}]}{dt} = k_f\ .\ [T7RNAP_{free}].\ [T7prGFP_{free}] - k_b\ .\ [PIC_{GFP}] - k_{init}\ .\ [PIC_{GFP}] \quad (5)$$

$$\frac{d[EC_{GFP}]}{dt} = k_{init}\ .\ [PIC_{GFP}] - \frac{k_{elong}}{L_{GFP}}\ .\ [EC_{GFP}] \quad (6)$$

$$\frac{d[mRNA_{GFP}]}{dt} = k_{init}\ .\ [PIC_{GFP}] \quad (7)$$

$$\frac{d[PIC_{T7RNAP}]}{dt} = k_f\ .\ [T7RNAP_{free}].\ [T7prT7RNAP_{free}] - k_b\ .\ [PIC_{T7RNAP}] - k_{init}\ .\ [PIC_{T7RNAP}] \quad (8)$$

$$\frac{d[EC_{T7RNAP}]}{dt} = k_{init}\ .\ [PIC_{T7RNAP}] - \frac{k_{elong}}{L_{T7RNAP}}\ .\ [EC_{T7RNAP}] \quad (9)$$

$$\frac{d[mRNA_{T7RNAP}]}{dt} = k_{init}\ .\ [PIC_{T7RNAP}] \quad (10)$$

Mole Balances

$$[T7pr_{total}] = [T7prT7RNAP_{free}] + [T7GFPpr_{free}] + [PIC_{GFP}] + [PIC_{T7RNAP}] \quad (11)$$

$$[T7RNAP_{total}] = [T7RNAP_{free}] + [PIC_{GFP}] + [EC_{GFP}] + [PIC_{T7RNAP}] + [EC_{T7RNAP}] \quad (12)$$

*Symbol descriptions and parameter values are listed in Supplementary Note 5.

Supplementary Note 3

Repressed Positive Feedback Loop (R-PFL)

ODEs

$$\frac{d[mRNA_{T7RNAP}]}{dt} = CopyN\ .\ (primingR_{T7RNAP} + R_{T7prT7RNAP}) - \delta_{mRNA}\ .\ [mRNA_{T7RNAP}] \quad (1)$$

$$\frac{d[T7RNAP]}{dt} = PR_{T7RNAP}\ .[mRNA_{T7RNAP}] - \delta_{T7RNAP}\ .[T7RNAP] \quad (2)$$

$$\frac{d[mRNA_{GFP}]}{dt} = CopyN\ .\ (leakyR_{GFP} + R_{T7prGFP}) - \delta_{mRNA}\ .[mRNA_{GFP}] \quad (3)$$

$$\frac{d[GFP]}{dt} = PR_{GFP}.[mRNA_{GFP}] - \delta_{GFP}\ .[GFP] \quad (4)$$

$$\frac{d[mRNA_{TetR}]}{dt} = CopyN\ .\ (leakyR_{TetR} + R_{J23100}) - \delta_{mRNA}\ .\ [mRNA_{TetR}] \quad (5)$$

$$\frac{d[TetR]}{dt} = PR_{TetR}\ .[mRNA_{TetR}] - \delta_{TetR}\ .[TetR] \quad (6)$$

Reaction Mechanism

$$\boldsymbol{T7RNAP_{free} + T7prGFP_{free} \underset{k_b}{\overset{k_f}{\leftrightarrows}} PIC_{GFP} \xrightarrow{k_{init}} EC_{GFP} \xrightarrow{k_{elong}} T7RNAP_{free} + T7prGFP_{free} + mRNA_{GFP}}$$

$$\boldsymbol{T7RNAP_{free} + T7tetOprT7RNAP_{free} \underset{k_b}{\overset{k_f}{\leftrightarrows}} PIC_{T7RNAP} \xrightarrow{k_{init}} EC_{T7RNAP} \xrightarrow{k_{elong}} T7RNAP_{free} + T7tetOprT7RNAP_{free} + mRNA_{T7RNAP}}$$

$$\boldsymbol{TetR_{free} + T7tetOprT7RNAP_{free} \leftrightarrow TetR{:}T7tetOprT7RNAP}$$

$$\frac{d[PIC_{GFP}]}{dt} = k_f\ .\left[T7RNAP_{free}\right].\left[T7prGFP_{free}\right] - \mathrm{k}_b\ .[PIC_{GFP}] - \mathrm{k}_{init}\ .[PIC_{GFP}] \quad (7)$$

$$\frac{d[EC_{GFP}]}{dt} = k_{init}\ .[PIC_{GFP}] - \frac{k_{elong}}{L_{GFP}}\ .[EC_{GFP}] \quad (8)$$

$$\frac{d[mRNA_{GFP}]}{dt} = k_{init}\ .[PIC_{GFP}] \quad (9)$$

$$\frac{d[PIC_{T7RNAP}]}{dt} = k_f\ .\left[T7RNAP_{free}\right].\left[T7tetOprT7RNAP_{free}\right] - \mathrm{k}_b\ .[PIC_{T7RNAP}] - \mathrm{k}_{init}\ .[PIC_{T7RNAP}] \quad (10)$$

$$\frac{d[TetR{:}T7tetOprT7RNAP]}{dt} = \frac{[TetR_{free}].[T7tetOprT7RNAP_{free}]}{K_d} \quad (11)$$

$$\frac{d[EC_{T7RNAP}]}{dt} = k_{init}\ .[PIC_{T7RNAP}] - \frac{k_{elong}}{L_{T7RNAP}}\ .[EC_{T7RNAP}] \quad (12)$$

$$\frac{d[mRNA_{T7RNAP}]}{dt} = k_{init}\ .[PIC_{T7RNAP}] \quad (13)$$

Mole Balances

$$[T7pr_{total}] = \left[T7pr_{free}\right] + [PIC_{GFP}] \quad (14)$$

$$[T7tetOpr_{total}] = \left[T7tetOpr_{free}\right] + [TetR{:}T7tetOprT7RNAP] + [PIC_{T7RNAP}] \quad (15)$$

$$[T7RNAP_{total}] = \left[T7RNAP_{free}\right] + [PIC_{GFP}] + [EC_{GFP}] + [PIC_{T7RNAP}] + [EC_{T7RNAP}] \quad (16)$$

$$[TetR_{total}] = \left[TetR_{free}\right] + [TetR{:}T7tetOprT7RNAP] \quad (17)$$

*Symbol descriptions and parameter values are listed in Supplementary Note 5.

Supplementary Note 4

Mixed (Negative+Positive) Feedback Loop (MFL)

ODEs

$$\frac{d[mRNA_{T7RNAP}]}{dt} = CopyN\ .\ (primingR_{T7RNAP} + R_{T7prT7RNAP}) - \delta_{mRNA}\ .\ [mRNA_{T7RNAP}] \quad (1)$$

$$\frac{d[T7RNAP]}{dt} = PR_{T7RNAP}\ .[mRNA_{T7RNAP}] - \delta_{T7RNAP}\ .[T7RNAP] \quad (2)$$

$$\frac{d[mRNA_{GFP}]}{dt} = CopyRK2\ .\ (leakyR_{GFP} + R_{T7prGFP}) - \delta_{mRNA}\ .[mRNA_{GFP}] \quad (3)$$

$$\frac{d[GFP]}{dt} = PR_{GFP}.[mRNA_{GFP}] - \delta_{GFP}\ .[GFP] \quad (4)$$

$$\frac{d[mRNA_{TetR}]}{dt} = CopyN\ .\ (leakyR_{TetR} + R_{T7prTetR}) - \delta_{mRNA}\ .\ [mRNA_{TetR}] \quad (5)$$

$$\frac{d[TetR]}{dt} = PR_{TetR}\ .[mRNA_{TetR}] - \delta_{TetR}\ .[TetR] \quad (6)$$

Reaction Mechanism

$$\boldsymbol{T7RNAP_{free} + T7prGFP_{free} \underset{k_b}{\overset{k_f}{\leftrightarrows}} PIC_{GFP} \xrightarrow{k_{init}} EC_{GFP} \xrightarrow{k_{elong}} T7RNAP_{free} + T7prGFP_{free} + mRNA_{GFP}}$$

$$\boldsymbol{T7RNAP_{free} + T7tetOprT7RNAP_{free} \underset{k_b}{\overset{k_f}{\leftrightarrows}} PIC_{T7RNAP} \xrightarrow{k_{init}} EC_{T7RNAP} \xrightarrow{k_{elong}} T7RNAP_{free} + T7tetOprT7RNAP_{free} + mRNA_{T7RNAP}}$$

$$\boldsymbol{T7RNAP_{free} + T7prTetR_{free} \underset{k_b}{\overset{k_f}{\leftrightarrows}} PIC_{TetR} \xrightarrow{k_{init}} EC_{TetR} \xrightarrow{k_{elong}} T7RNAP_{free} + T7prTetR_{free} + mRNA_{TetR}}$$

$$\boldsymbol{TetR_{free} + T7tetOprT7RNAP_{free} \leftrightarrow TetR{:}T7tetOprT7RNAP}$$

$$\frac{d[PIC_{GFP}]}{dt} = k_f\ .[T7RNAP_{free}].[T7prGFP_{free}] - \mathrm{k}_b\ .[PIC_{GFP}] - \mathrm{k}_{init}\ .[PIC_{GFP}] \quad (7)$$

$$\frac{d[EC_{GFP}]}{dt} = k_{init}\ .[PIC_{GFP}] - \frac{k_{elong}}{L_{GFP}}\ .[EC_{GFP}] \quad (8)$$

$$\frac{d[mRNA_{GFP}]}{dt} = k_{init}\ .[PIC_{GFP}] \quad (9)$$

$$\frac{d[PIC_{T7RNAP}]}{dt} = k_f\ .[T7RNAP_{free}].[T7tetOprT7RNAP_{free}] - \mathrm{k}_b\ .[PIC_{T7RNAP}] - \mathrm{k}_{init}\ .[PIC_{T7RNAP}] \quad (10)$$

$$\frac{d[TetR{:}T7tetOprT7RNAP]}{dt} = \frac{[TetR_{free}].[T7tetOprT7RNAP_{free}]}{K_d} \quad (11)$$

$$\frac{d[EC_{T7RNAP}]}{dt} = k_{init}\ .[PIC_{T7RNAP}] - \frac{k_{elong}}{L_{T7RNAP}}\ .[EC_{T7RNAP}] \quad (12)$$

$$\frac{d[mRNA_{T7RNAP}]}{dt} = k_{init}\ .[PIC_{T7RNAP}] \quad (13)$$

$$\frac{d[PIC_{TetR}]}{dt} = k_f\ .[T7RNAP_{free}].[T7prTetR_{free}] - \mathrm{k}_b\ .[PIC_{TetR}] - \mathrm{k}_{init}\ .[PIC_{TetR}] \quad (14)$$

$$\frac{d[EC_{TetR}]}{dt} = k_{init}\ .[PIC_{TetR}] - \frac{k_{elong}}{L_{GFP}}\ .[EC_{TetR}] \quad (15)$$

$$\frac{d[mRNA_{TetR}]}{dt} = k_{init}\ .[PIC_{TetR}] \quad (16)$$

Mole Balances

$$[T7pr_{total}] = [T7prT7RNAP_{free}] + [T7GFPpr_{free}] + [PIC_{GFP}] + [PIC_{T7RNAP}] \quad (17)$$

$$[T7tetOpr_{total}] = [T7tetOpr_{free}] + [TetR{:}T7tetOprT7RNAP] + [PIC_{T7RNAP}] \quad (18)$$

$$[T7RNAP_{total}] = [T7RNAP_{free}] + [PIC_{GFP}] + [EC_{GFP}] + [PIC_{T7RNAP}] + [EC_{T7RNAP}] + [PIC_{TetR}] + [EC_{TetR}] \quad (19)$$

$$[TetR_{total}] = [TetR_{free}] + [TetR{:}T7tetOprT7RNAP] \quad (20)$$

*Symbol descriptions and parameter values are listed in Supplementary Note 5.

Supplementary Note 5

The symbols in Supplementary Notes 1-4 represent the following entities:

$\text{priming}R_{T7RNAP}$, $\text{leaky}R_{GFP}$ and $\text{leaky}R_{TetR}$ represent basal transcription rates (nM min^{-1}),
$R_{T7prT7RNAP}$, $R_{T7prGFP}$ and $R_{T7prTetR}$ represent transcription rates from the T7pr (nM min^{-1}),
R_{J23100} represents transcription rate from the J23100 pr (nM min^{-1}),
L_{T7RNAP}, L_{GFP} and L_{TetR} represent lengths of mRNAs (nt),
$mRNA_{T7RNAP}$, $mRNA_{GFP}$ and $mRNA_{TetR}$ represent mRNA concentrations (nM),
δmRNA represents the mRNA degradation rate set to a constant of log(2)/5 min^{-1},

PR_{T7RNAP}, PR_{GFP} and PR_{TetR} represent protein translation rates (min^{-1}),
T7RNAP, GFP and TetR represent the total protein concentrations (nM),
$T7RNAP_{free}$ represents the unbound T7RNAP (nM),
$TetR_{free}$ represents the unbound TetR (nM),
δ_{T7RNAP}, δ_{GFP} and δ_{TetR} represent protein degradation rates (min^{-1}),

$T7pr_{total}$ represents the total T7 promoter concentration (nM),
$T7tetOpr_{total}$ represents the total T7-tetO promoter concentration (nM),
$T7prT7RNAP_{free}$, $T7prGFP_{free}$ and $T7prTetR_{free}$ represent free T7 promoter concentrations (nM),

PIC_{T7RNAP}, PIC_{GFP} and PIC_{TetR} represent T7RNAP-driven transcription pre-initiation complex concentrations (nM),
k_b represents the T7RNAP backward rate constant (min^{-1}),
k_f represents the T7RNAP forward rate constant (min^{-1} nM^{-1}),
k_{init} represents the T7RNAP-driven transcription initiation rate constant (min^{-1}),
EC_{T7RNAP}, EC_{GFP} and EC_{TetR} represent T7RNAP-driven transcription elongation complex concentrations (nM),
k_{elong} represents the T7RNAP-driven transcription elongation rate constant (nt min^{-1})
Kd represents the TetR dissociation constant (nM),

CopyN represents the UBER plasmid copy number per cell in all systems,
CopyRK represents the GFP expression plasmid copy number per cell in the MFL system.

The various parameter values used in the four systems are listed below:

**Parameter**	**Value**	**Units**
CopyN	51.49	-
k_{init}	0.0015	min^{-1}
k_b	0.0003311	min^{-1}
k_f	0.000131	min^{-1} nM^{-1}

k_{elong}	16293.9638	nt min^{-1}
δ_{T7RNAP}	0.000963+μ	min^{-1}
δ_{GFP}	0.000481+μ	min^{-1}
PR_{T7RNAP}	$(RBS/10^5)*0.017608$	min^{-1}
$PR_{GFP}*Flu_{PC}$	$(RBS/10^5)*0.22871$	min^{-1}
Ptox	0.123	-

Where,

μ represents the specific growth rate (min^{-1}),

RBS represents translation initiation rate (RBS Calculator v2.0 units),

Lumped together with PR_{GFP}, Flu_{PC} represents a proportionality constant that converts cellular GFP concentrations to Fluorescence (au),

For steady state model solutions, all protein translation rates (PR_{T7RNAP}, PR_{GFP} and PR_{TetR}) are multiplied by a factor of $1/(1 + T7RNAP^{Ptox})$ to account for reduction in global translation due to T7 RNAP toxicity.

The following parameters are system-specific, and their values may vary from one system to another:

**Parameter**	**PFL-**	**PFL+**	**R-PFL**	**MFL**	**Units**
$primingR_{T7RNAP}$	0.020877	0.386238	0.32144	5.579613	min^{-1}
$primingR_{T7RNAP}$ (HIGH)	46.454163	50.897095	-	-	min^{-1}
$leakyR_{GFP}$	0.00361	0.001702	0.001941	0.000025	min^{-1}
R_{J23100}	-	-	6.162056	-	min^{-1}
$leakyR_{TetR}$	-	-	-	0.818529	min^{-1}
Kd	-	-	166.979225	166.979225	nM
δ_{TetR}	-	-	0.030597 (+ssRA)	0.001463 (-ssRA)	min^{-1}
PR_{TetR}	-	-	3.846874	3.846874	min^{-1}
CopyRK	-	-	-	3.48	-

Where experimental data is unavailable, Specific Growth Rates (μ) used for model simulations were calculated as a function of T7 RNAP RBS. Estimating from experimental data (Figure 3B), for PFL- system $\mu=0.9679*e^{-RBS/10^5}$ hr^{-1} ($R^2=0.8436$), while for PFL+ system $\mu=0.8227*e^{-RBS/10^4}$ hr^{-1} ($R^2=0.9777$).

Supplementary Note 6

Parameter values in Supplementary Note 5 were estimated by following a six step fitting process, using fminsearch (MATLAB, Mathworks) to minimize the model's error functions:

Step-1

- Closed-loop Positive Feedback Loop (PFL+) model was fitted using the time course GFP fluorescence data for T7 RNAP RBSes 328, 1156 and 2244 (Figure 3C). RBS 9158 was excluded because those cells had GFP fluorescence much higher than basal level and remained in a prolonged lag-phase until 335 min, presumably due to toxicity from the residual pre-chill T7 RNAP (see growth curve below).
- An initial condition of [$mRNA_{T7RNAP}$=0, T7RNAP=0, $mRNA_{GFP}$=0, GFP=0] was used for all simulations with some assumptions: (1) most protein production has been stopped by chilling of inoculant cultures, (2) any pre-chill residual levels of mRNA and protein within the inoculant culture will be diluted by cell division, and (3) the amount of the pre-chill residual level of mRNA or protein will be low and will not significantly affect the model fitting. These assumptions seem to hold well for RBS variants 328, 1156 and 2244 where GFP fluorescence at the initial time points is very close to basal level.
- Only data from the first eight time points (110, 150, 205, 255, 285, 335, 380 and 430 min) before the cells entered stationary phase were used for fitting each RBS variant, and the doubling time was set to 40 min.

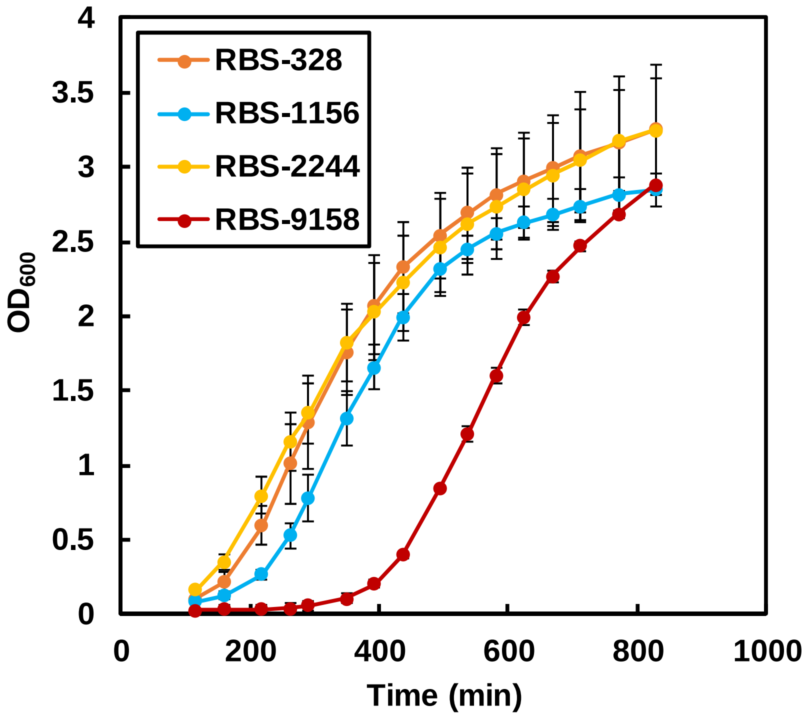

- Two parameters were manually set (δ_{T7RNAP} and δ_{GFP}), and ten parameter values were estimated in this step of fitting (33.4% average error)– CopyN, k_{init}, k_b, k_f, k_{elong}, PR_{T7RNAP}, PR_{GFP}* Flu_{PC}, $primingR_{T7RNAP}$, $leakyR_{GFP}$, and δmRNA. Error function used was |Δx|/x.

- A sensitivity analysis was carried out, and six of the ten parameters were found to be most sensitive for the model solution– k_{init}, k_f, PR_{T7RNAP}, PR_{GFP}* Flu_{PC}, primingR_{T7RNAP}, and δmRNA. These were re-fitted to the same data, obtaining a best-fit with 16.2% average error.

Step-2

- The effect of T7 RNAP toxicity was modelled using Hill Function kinetics, multiplying all protein translation rates by a factor of $1/(1+T7RNAP^{Ptox})$ to account for reduction in global translation. The Positive Feedback Loop (PFL+) model described above was modified to include the toxicity parameter Ptox, and its value was estimated by fitting to steady state GFP fluorescence data and Specific Growth Rates (Figure 3B, right), using the error function |Δ log x|/log x.
- Four of the five RBSes were fitted at a time. For each fitting, average error of the fitted RBS set as well as that of the excluded RBS were reported for cross-validation.

**Fit #**	**RBS excluded**	**4-RBS Avg. Error (%)**	**Excluded RBS error (%)**	**Parameter Values**
				**Ptox**
1	28	1.80	23.49	0.236334
2	328	3.74	10.48	0.068539
3	1156	7.06	1.18	**0.122637**
4	2244	5.81	6.07	0.11852
5	9156	5.42	7.69	0.120636

- Parameter values from Fit #3 were chosen because that showed the least error for the excluded RBS, though the best-fit Ptox values did not significantly vary during cross-validation.

Step-3

- The ten parameter values obtained above were plugged into the Open Loop (PFL-) model, and two system-specific parameters (primingR_{T7RNAP} and leakyR_{GFP}) were estimated by fitting to steady state GFP fluorescence data and Specific Growth Rates (Figure 3B, left) , using the error function |Δ log x|/log x.
- Four of the five RBSes were fitted at a time. For each fitting, average error of the fitted RBS set as well as that of the excluded RBS were reported for cross-validation.

**Fit #**	**RBS excluded**	**4-RBS Avg. Error (%)**	**Excluded RBS error (%)**	**Parameter Values**	
				**primingR_{T7RNAP}**	**leakyR_{GFP}**
1	97	0.85	15.55	0.009519	0.007924
2	2171	0.93	6.08	0.019406	0.004147
3	5748	1.93	0.88	**0.020877**	**0.00361**
4	7656	1.99	0.91	0.02302	0.003622
5	21439	1.84	1.52	0.023068	0.003621

- Parameter values from Fit #3 were chosen because that showed the least error for the excluded RBS, though the best-fit parameter values did not significantly change during cross-validation.

Step-4

- primingR_{T7RNAP} value for the High Priming pr version of the PFL- and PFL+ systems was estimated by fitting the models to one data-point each of steady state GFP fluorescence data and Specific Growth Rate (Figure 1B, two middle bars), using the error function $|\log \Delta x|/\log x$. Average errors for the PFL- and PFL+ systems were 1.85% and 5.00%, respectively.

Step-5

- Other parameter values obtained above were plugged into the Repressed Positive Feedback Loop (R-PFL) model, and six system-specific parameters (primingR_{T7RNAP}, leakyR_{GFP}, R_{J23100}, Kd, δ_{TetR}, and PR_{TetR}) were estimated by fitting to steady state GFP fluorescence data and Specific Growth Rates (Figure 4C), using the error function $|\Delta \log x|/\log x$.
- Data from RBSes 328, 1156, 2244 and 9158 were fitted together with an average error of 2.26%.

Step-6

- Other parameter values obtained above were plugged into the Mixed Feedback Loop (MFL) model, and five system-specific parameters (primingR_{T7RNAP}, leakyR_{GFP}, leakyR_{TetR}, δ_{TetR}, and CopyRK) were estimated by fitting to steady state GFP fluorescence data and Specific Growth Rates (Figure 5B), using the error function $|\Delta \log x|/\log x$.
- Data from all the 15 clones were fitted together with an average error of 11.51%.

Supplementary References

1. Waibel, A., Hanazawa, T., Hinton, G., Shikano, K. & Lang, K. J. Phoneme recognition using time-delay neural networks. *IEEE Trans. Acoust.* **37,** 328–339 (1989).

2. Solovyev, V. & Salamov, A. Automatic annotation of microbial genomes and metagenomic sequences. *Metagenomics its Appl. Agric. Biomed. Environ. Stud. (Ed. RW Li), Nov. Sci. Publ.* 61–78 (2011).

3. Salis, H. M. The ribosome binding site calculator. *Methods Enzymol.* **498,** 19–42 (2011).

4. Temme, K., Hill, R., Segall-Shapiro, T. H., Moser, F. & Voigt, C. a. Modular control of multiple pathways using engineered orthogonal T7 polymerases. *Nucleic acids research*, 1-9, doi:10.1093/nar/gks597 (2012).
